# Memory CD4+ T cells sequentially restructure their 3D genome during stepwise activation

**DOI:** 10.3389/fcell.2025.1514627

**Published:** 2025-02-13

**Authors:** Alexander I. Ward, Jose I. de las Heras, Eric C. Schirmer, Ariberto Fassati

**Affiliations:** ^1^ Institute of Immunity and Transplantation, Division of Infection and Immunity, University College London, London, United Kingdom; ^2^ Institute of Cell Biology, University of Edinburgh, Edinburgh, United Kingdom

**Keywords:** 3D-genome organization, memory CD4+ T cells, sequential immune activation, gene expression regulation, Hi-C, IL-2, enhancer–promoter interactions, primed/de-primed genes

## Abstract

**Background:**

CD4+ T cells are a highly differentiated cell type that maintain enough transcriptomic plasticity to cycle between activated and memory statuses. How the 1D chromatin state and 3D chromatin architecture support this plasticity is under intensive investigation.

**Methods:**

Here, we wished to test a commercially available *in situ* Hi-C kit (Arima Genomics Inc.) to establish whether published performance on limiting cell numbers from clonal cell lines copies across to a primary immune cell type. We achieved comparable contact matrices from 50,000, 250,000, and 1,000,000 memory CD4+ T-cell inputs. We generated multiple Hi-C and RNA-seq libraries from the same biological blood donors under three separate conditions: unstimulated fresh *ex vivo*, IL-2-only stimulated, and T cell receptor (TCR)+CD28+IL-2-stimulated, conferring increasingly stronger activation signals. We wished to capture the magnitude and progression of 3D chromatin shifts and correlate these to expression changes under the two stimulations.

**Results:**

Although some genome organization changes occurred concomitantly with changes in gene expression, at least as many changes occurred without corresponding changes in expression. Counter to the hypothesis that topologically associated domains (TADs) are largely invariant structures providing a scaffold for dynamic looping contacts between enhancers and promotors, we found that there were at least as many dynamic TAD changes. Stimulation with IL-2 alone triggered many changes in genome organization, and many of these changes were strengthened by additional TCR and CD28 co-receptor stimulation.

**Conclusions:**

This suggests a stepwise process whereby mCD4+ T cells undergo sequential buildup of 3D architecture induced by distinct or combined stimuli likely to “prime” or “deprime” them for expression responses to subsequent TCR-antigen ligation or additional cytokine stimulation.

## Introduction

CD4+ T cells are critical to orchestrating adaptive immune responses, and their loss, typically observed in untreated HIV-1 infection, causes profound immune deficiency (Simon et al., 2006). CD4+ T cells can be broadly classified as naïve or memory. Memory CD4+ (mCD4) T cells derive from naïve cells following MHC-II-presented antigen stimulation and exposure to co-stimulation and maintain the ability to respond to the specific antigen for decades (Ahmed and Gray, 1996; Farber et al., 2014; Raphael et al., 2020). Whereas in peripheral blood, mCD4+ T cells are mostly in a quiescent state with only occasional homeostatic cell division (Rufer et al., 2001), upon re-encountering their cognate antigen, these cells rapidly switch to an activated or effector state (the “recall” response) characterized by rapid cell division, high metabolic activity, and secretion of cytokines that ultimately lead to the recruitment of additional immune cells and elimination of the target pathogen (Pepper and Jenkins, 2011; Williams and Bevan, 2007). mCD4+ T cells can be classified into central memory and effector memory, which may differentiate into four main functional subsets: T helper (Th)-1, secreting cytokines IFNγ, and tumor necrosis factor (TNF) and protecting against intracellular viruses and bacteria; Th2, secreting IL-4, IL-5, IL-10, and IL-13 and protecting against extracellular helminths; Th17, secreting IL-17 and protecting against extracellular fungi and bacteria; and T-regulatory (Treg), secreting IL-10 and TGFβ and protecting against excessive immune inflammation and autoimmunity (Farber et al., 2014; Pepper and Jenkins, 2011; Luckheeram et al., 2012).

The development of global techniques to analyze the 3D genome interactions at high resolution, such as Hi-C, revealed some key organizing features (Bonev and Cavalli, 2016). Principle component analysis (PCA) of the Hi-C interaction matrix segregated two distinct spatial genomic subtypes, called A and B compartments, based on their tendencies to have fewer reciprocal interactions. Further correlations with histone marks and gene expression data indicated that the A compartment is highly enriched for active genes while the B compartment is enriched for inactive genes. Intersection of Hi-C data with another spatial genome approach called DamID that has been used to distinguish genome regions at the nuclear periphery (termed LADs for lamina-associated domains) from those in the nuclear interior (Pickersgill et al., 2006) indicated that most of the genome located at the nuclear periphery is in the B compartment (Zheng et al., 2018).

Within these large compartments, a high density of local interactions within specific chromosome regions <1 Mb in size define topologically associated domains or TADs. Although, for the most part, TADs tend to be invariant across cell types, smaller clusters within TADs, called sub-TADs (Cao et al., 2023), have been shown to be more dynamic (Hansen et al., 2018), and less cell-type invariant (Tan et al., 2023). Sub-TADs change conformation in response to signaling cascades, driving transcriptional responses to stimuli in differentiated cells (Winick-Ng et al., 2021) and in cell-fate decisions during development (Boltsis et al., 2021). Often, distinct chromatin regions are tethered by chromatin-associated proteins such as CTCF, cohesin, or condensin, forming chromatin fiber extrusions called loops (Grubert et al., 2020). Loops include enhancer-promoter contacts and are delimited by boundaries at the sites of CTCF and cohesin binding. Thus, they provide a mechanism for spatial genome organization to influence gene expression (Ron et al., 2017).

However, the functional connection between patterns of genome spatial organization and regulation of gene expression is only partially established, and ablation of CTCF or cohesin causes widespread loss of TAD boundaries and impairs loop formation (Grubert et al., 2020; Haarhuis et al., 2017; Nora et al., 2017) but does not appear to cause global dis-regulation of gene expression. There are several possible explanations for these results. Genome 3D organization may have multiple distinct roles in transcriptional regulation, with some interactions acting as barriers, while others might “prime” or “poise” inactive genes for activation once transcription factors (TFs) are available, and yet others could facilitate cell-fate conversions with only a subset of spatial interactions actively optimizing gene expression (Stadhouders et al., 2019). Furthermore, a DamID study of T-cell activation using the Jurkat CD4+ T-cell line revealed that many genes important for activation are released from LADs at the nuclear envelope into the A compartment yet remain peripheral, suggesting that re-recruitment back to the nuclear envelope could be a mechanism for tempering immune responses (Robson et al., 2017).

The 1D (Rothenberg, 2014) epigenetic and 3D (Hu et al., 2018) architectural drivers of expression changes on T-cell activation have been intensely investigated. During T-cell development in the thymus, sequential bursts of mobilization of lineage-defining TFs Bcl11b and TCF1 modify the baseline chromatin state to suppress alterative lineages and activate enhancers driving expression of the machinery necessary for T-cell receptor (TCR) expression and assembly. Many of the principles of chromatin state control are evident in thymic development, including non-coding RNA-directed CTCF binding (Isoda et al., 2017), metabolic integration of epigenetic code deposition (Mocholi et al., 2023), and enhancer hubs driving the continued expression of lineage-defining genes (Zelenka et al., 2022). Mature naïve CD4+ T cells are equipped to respond to various cytokines via diverse STAT signaling cascades that pivot the final differentiated effectors through expression of master TFs T-bet (Th1), GATA3 (Th2), ROR-γt (Th17), and FOXP3 (Tregs). A heritable bias in differentiation potential has been reported to be encoded as 1D inherited chromatin marks (Rogers et al., 2021) that modulate sensitivity to incoming TFs. Whether this extends to the calls of 3D chromatin contacts is unknown. Naïve T cells are released into the periphery for Ag encounter and immune response. FOXP3 has been shown to be a supervisor of functional chromatin contacts (Liu et al., 2023) in Treg cells. Along with the polarization of effector cytokine output, CD4+ T cells must generate both large populations of terminally differentiated effectors during Ag encounters along with a smaller pool of memory cells that both retain a high proliferative capacity and Ag-independent viability. This is accomplished by gene regulatory circuitry that resists, either with a pre-bias or stochastically, a complete switch away from the naïve/memory stem-like 1D/3D chromatin state and outputted transcriptome (Russ et al., 2023; Quon et al., 2023; Wang et al., 2022). This spectrum of maintenance of stemness vs. terminal differentiation could form the basis of the more rapid proliferative and secretory phenotype seen in memory populations (Onrust-van Schoonhoven et al., 2023; Zhu et al., 2023).

We wished to apply deep Hi-C sequencing to capture these shifts in 3D chromatin contacts in this terminal stage of CD4+ T-cell fate and correlate these with RNA abundance in the transcriptome (Bediaga et al., 2021). By analyzing and comparing unstimulated cells to those stimulated with IL-2 only or IL-2 plus T-cell receptor (TCR) and CD28 co-receptor activation, we obtained highly reproducible maps of comparative quality and resolution from 1 million, 250,000, and 50,000 mCD4+ T cells from a single donor using the commercially available *in situ* Hi-C kit (Arima Genomics Inc.). We found that the 3D architecture of chromatin at important immune loci builds up in a sequential manner in CD4+ T cells upon a stepwise increase in immune stimulation. We also show that, at this unreported sequencing depth on a single biological mCD4+ T-cell donor, most of the genic loci that change expression do not overlap with those undergoing significant alterations in 3D chromatin organization.

## Materials and methods

### Isolation and activation of mCD4+ T cells

**FIGURE 1:**
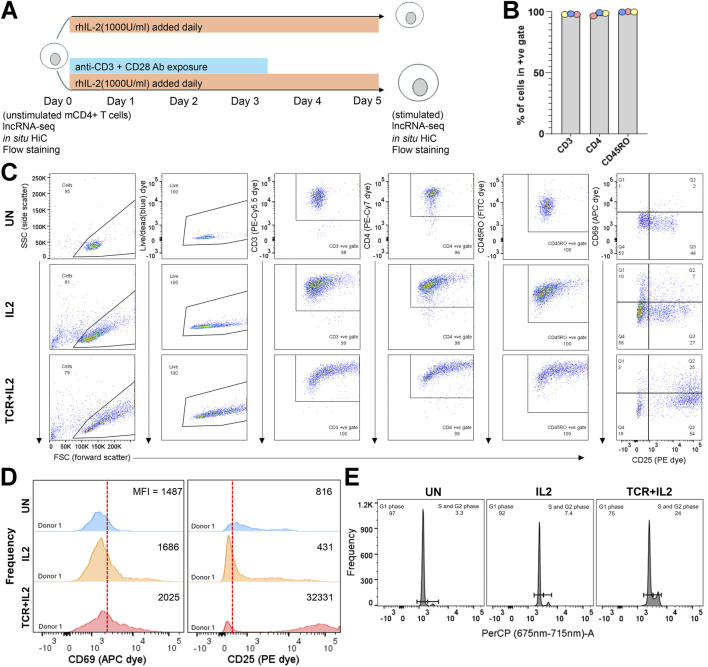
Isolation and stimulation of mCD4-T cells for RNA-seq and *in situ* Hi-C assays. **(A)** Cartoon of *ex vivo* stimulation of mCD4-T cells showing populations on which genomic assays were done. **(B)** Percentage of events in the unstimulated populations across the three donors falling into the +ve gates for each dye; see Supplementary Figure S1 for the fluorescence minus one (FMO) gate setting and the raw flow dot plots. The isolated CD4m-cell population had excellent purity for CD3, CD4, and CD45RO expression. **(C)** Donor 1 unstimulated, TCR + IL2, and IL2 populations flow dot plots stained with the full panel of dyes; see Supplementary Figure S1, S2 for donor 2 and 3 data (a plot to gate on singlets is excluded, >99% of cellular events were singlets). **(D)** Histograms showing the frequency of each bin of CD69 (APC dye) and CD25 (PE dye) expression for donor 1 in the unstimulated TCR + IL2 and IL2-stimulated populations. The vertical red line separates the CD25/CD69 negative (left) from the positive (right) population as placed by FMO gating (MFI, median fluorescence intensity). Donor 2 and 3 data are shown in Supplementary Figure S2. **(E)** Propidium iodide staining for cell cycle phase assessment of donor 1 populations. The signal was captured using the PerCP detector. Histograms for donors 2 and 3 are shown in Supplementary Figure S3.

Healthy human donor blood cones were ordered from NHS Blood and Transplant Services, United Kingdom, under UCL Research Ethics Committee Approval reference 3,138/001. This agreement ensures donor anonymity, so donations do not come with any demographic information. The contents of each cone were diluted in 8 mL 1 × PBS and layered onto 15 mL Ficoll in X4 50 mL tubes. Tubes were subjected to centrifugation at 2000 rpm for 20 min at RT. Peripheral blood mononuclear cells (PBMCs) were aspirated using a transfer pipette and washed twice in 10 mL 2 mM EDTA 1 × PBS in 15 mL conical tubes (350 × g 5 min at room temperature, RT). A third wash was done at 200 × g 5 min at RT to maximize platelet removal. PBMCs were spun at 350 × g and resuspended in x1 Mojosort buffer (1 × PBS, 2 mM EDTA, 0.5% BSA) and diluted to 1 × 10^8^ PMBCs/mL. All cell counts were done using an OLS CASY cell counter. mCD4+ T cells were isolated by magnetic separation Biolegend (480,064) following the manufacturer’s protocol. Purity was assessed by flow cytometry (Figure 1; Supplementary Figures S1, S2). Isolated T cells were activated at 2.5 × 10^6^/ml in RPMI 10% FBS 0.5% penicillin/streptomycin solution (Gibco, 15,070,063). The base of 24-well culture dishes was treated for 45 min with 1 mL 1 × PBS containing anti-CD3 antibody Biolegend (317,326) clone OKT at 476 ng/mL. Plates were washed once with 1 × PBS. On day 0 only, anti-CD28 antibody Biolegend (302,902) clone CD28.2 at 1,000 ng/mL was added. T cells were allowed to expand in wells for three days with 1,000 U/mL IL-2 (Prepotech, 200–02) added daily. For days 4 and 5, T cells were transferred to T25 untreated culture flasks with additional RPMI added to prevent medium exhaustion (assessed by color change from pink to yellow). On each of these days, 1000 U/mL IL-2 was added.

### Flow cytometry

Fluorescent antibody panel (Live/Dead (blue), L23105), CD3 Biolegend (317,336), CD4 Biolegend (317,414), CD45RO Biolegend (304,204), CD69 Biolegend (310,910), and CD25 Biolegend (302,606) was validated for specificity using fluorescence minus one (Supplementary Figures S1). For this, 3 × 10^5^ cells were pelleted at 4°C, 350 × g, for 5 min and suspended in 100 μL 1x PBS 0.001% Live/Dead (blue) dye. A 1 μL aliquot of each fluorescent antibody was added and briefly vortexed before incubation at 4°C for 30 min. Stained cells were spun and washed once with 0.5 mL ice-cold 1xPBS 1% FBS 2 mM EDTA 0.01% sodium azide, with final suspensions in 300 μL. Samples were run on a BD LSR Fortessa with baseline voltage setting (except CD25, for which the voltage was reduced by 50 units) with gating and analysis done in FlowJo v_10.8.1.

### RNA extraction

Cells were pelleted and frozen in freezing buffer (fetal bovine serum 10% DMSO) at −70°C. Total RNA was extracted from each sample, and a high molecular weight fraction (>200 nt) was isolated using the Qiagen RNeasy (74,134) kit with on-column DNase treatment (79,254), according to the manufacturer’s instructions. Final elutions were done at 50 µL NFW and were snap frozen on dry ice. RNA quality was assessed by Novogene (United Kingdom) with a Bioanalyzer (Agilent Technologies), and all samples with an RNA integrity number (RIN) score >5.5 and mass >500 ng were forwarded to Novogene’s lncRNA-seq protocol for library prep and sequencing (Supplementary Table S1).

### Differential gene expression analysis

On average, 45 million paired-end (PE) fragments were sequenced per technical replicate. Donor 1 had three replicates, while donors 2 and 3 had two each (Supplementary Table S1). Sequencing quality was assessed with FastQC v0.11.9 (https://www.bioinformatics.babraham.ac.uk/projects/fastqc). Sequencing adaptors were removed with Trimmomatic v0.35 (Bolger et al., 2014). Low-quality reads and mitochondrial contaminants were removed. Differential expression analysis was performed in R with DESeq2 v1.32.0 (Love et al., 2014) after transcript quantitation with Salmon v1.4.0 (Patro et al., 2017). We used an FDR of 1% and 2-fold change thresholds for differential expression (Supplementary Table S2).

### Functional analysis of gene sets

Functional analyses were performed with g:Profiler (Reimand et al., 2016). g:Profiler was used to determine enriched categories within a set of DE genes, with an FDR of 5% as threshold. To determine the top terms, the GO Biological Process terms were first sorted for enrichment over the expected based on the number of associated genes changing in the condition. As there is considerable redundancy amongst GO-terms, after this initial sorting, similar terms were removed—particularly those that were a subset of a larger term—so that the variety of genes changing could be better viewed. For example, the cell cycle dominated the GO-terms, so both multiple terms for essentially the same function, such as “spindle pole assembly” and “kinetochore microtubule attachment” would have redundant ones deleted, but also because this is all part of cell division along with many other aspects, multiple sub-parts of the process would be removed to retain only one more-encompassing term. Note that in making these decisions, redundancy amongst the gene lists was also considered. The complete unfiltered results are shown in Supplementary Table S3.

### *In situ* Hi-C (Arima Genomics kit)

Cells were counted and spun into pellets of differing sizes at 5000 × g for 5 min. The supernatant was removed until <20 µl of liquid + cell pellet remained. The pellets were snap frozen on dry ice and then stored at −70°C until processing. All pellets were committed to the mammalian cell low-input Arima Genomics Inc. workflow (https://arimagenomics.com/wp-content/files/User-Guide-Arima-Hi-C-for-Mammalian-Cell-Lines.pdf, September 2024) with the following modifications: conditioning incubation time 20 min, restriction enzyme cutting time 1 h. Proximally ligated DNA samples were quality-controlled (QC) for biotin nucleotide incorporation if enough material was available. Samples that failed QC were not advanced further. Sonication was done using an S220 Covaris machine (Imperial College London) with company-recommended settings (pn_010368.pdf (covaris.com), September 2024) to achieve ∼400 bp fragment peaks. Fragment distribution was assessed using an Agilent TapeStation 4150 using a dsDNA D1000 or D5000 cartridge. Library preparation was done by inputting material into the Arima Genomics library prep module (Microsoft Word - User Guide Arima Library Prep for Arima-Hi-C+.docx (arimagenomics.com), September 2024). The final libraries were quality controlled before shipment by Qubit dsDNA assay and TapeStation as above. Libraries were submitted for sequencing to Novogene (United Kingdom) in the pre-made DNA workflow to generate ∼90 Gb 150 bp PE reads per sample. fastq files were downloaded for entry into the Bioinformatics workflow (Supplementary Table S4).

### Hi-C bioinformatic analysis

Sequences were aligned to the Hg38 genome (Ensembl v106) Bowtie2, QC-checked, and valid contact pairs in BAM format extracted using HICUP 0.8.3. Most analyses used tools from HOMER (Heinz et al., 2010) and Hi-C Explorer (Wolff et al., 2020). Around 300 million pair-end reads were obtained from each sample derived from the three donors. Donor 1 was sequenced in duplicate for each treatment (UN, IL2, and TCR + IL2) and input cell number (50 k, 250 k, and 1000 k cells). Donors 2 and 3 were employed in order to confirm that the patterns observed in the donor 1 data were general and not particular to that donor. Once the reproducibility of the data from the different inputs and replicates was established with Hi-C Explorer hicCorrelate, these were pooled. This resulted in ∼0.3B valid contacts in duplicate or ∼0.6B valid contacts per condition with the pooled replicates. Pooled replicates were used for the discovery of genomic features, while the separate replicates were used for the assessment of statistical significance (Supplementary Table S4). The number of contacts and other metrics were obtained with Hi-C Explorer hicInfo.

A and B compartments were calculated with HOMER runHiCpca at 40 kb resolution, and compartment switches were identified with HOMER getHiCcorrDiff. Four A and four B subcompartments were calculated using CALDER (Liu et al., 2021). TADs were detected using HOMER findTADsAndLoops using a range of resolution parameters (3 kb, 5 kb, 10 kb, 25 kb, 50 kb, 100 kb, and 200 kb) and window sizes 5× that of the resolution, with a search space of 5 Mb. A consensus set of TADs was derived considering TADs from all treatments and parameterizations. Loops were detected with HiC Explorer hicDetectLoops with –peakWidth values of 4, 5, and 30, and –windowSize 10, 20, and 60, respectively, in order to capture small sharp loops as well as larger diffused ones. Differential TADs and loops were identified using the HOMER score and HOMER getDiffExpression, which employs edgeR (Robinson et al., 2010) to calculate the statistical significance.

Enhancer-promoter associations were identified using a set of 4,252 enhancers for human CD4+ T cells from Enhancer Atlas 2.0 (Gao and Qian, 2020) and promoters from the gene set in the Ensembl Hg38 genome, version v106. A promoter was defined as a region between 2 kb upstream and 1 kb downstream of each transcriptional start site. In brief, a virtual 4C approach was employed using the enhancer set with HiContacts (Serizay et al., 2024) to detect interactions and HOMER analyzeHiC -interactions to assign statistical significance to each enhancer interaction, using an FDR of 5% as a threshold. Next, each positive interaction was matched to the nearest promoter with BEDTools closest (Quinlan and Hall, 2010). Differential enhancer-promoter interactions were identified using the HOMER score and HOMER getDiffExpression, which employs edgeR (Robinson et al., 2010) to calculate the statistical significance. Visualization of Hi-C contact maps was performed by extracting normalized Hi-C matrices at 5 kb resolution and a 20 kb smoothing window using HOMER analyzeHiC and plotting them with a custom script based on R package pheatmap v1.0.12. Genomic annotation tracks were visualized with IGV (Robinson et al., 2011).

## Results

### Isolation and stimulation of mCD4+ T cells

To investigate the relationship between 3D chromatin conformation and mCD4+ T-cell activation, we purified this cell population from peripheral blood mononuclear cells (PBMCs) of three healthy donors using magnetic isolation and performed parallel RNA-seq and *in situ* Hi-C (Ramani et al., 2016). Flow cytometry was used to examine the purity of the mCD4+ T-cell population based on the well-established markers CD3, CD4, and CD45RO (Mousset et al., 2019). Positive gates were established by staining with the 6-dye panel minus one (Supplementary Figure S1). This magnetically sorted cell population was divided into three aliquots. One aliquot was left untreated (unstimulated or “UN”), one aliquot was treated with IL-2 (1000 U/mL) added daily for 5 days (hereafter termed “IL2”), and another aliquot was similarly treated with IL-2 but also with anti-CD3 (TCR) and CD28 (co-stimulatory receptor) monoclonal antibodies for the first 72 h in culture (hereafter defined as “IL2+TCR”) (Figure 1A). The addition of the IL-2 alone supports mCD4 T-cell survival and proliferation (Vella et al., 1998) and promotes the differentiation of mCD4+ T cells toward the Treg, Th1, and Th2 lineages but represses the differentiation of the pro-inflammatory Th17 lineage (Chinen et al., 2016; Almeida et al., 2002). The treatment with the antibodies clusters both the T-cell receptor (TCR) and the CD28 co-stimulator molecules and triggers maximal stimulation (Trickett and Kwan, 2003). RNA was extracted from the unstimulated population on day 0 and from the IL2 and IL2+TCR populations on day 5. Cells were pelleted and fixed with formaldehyde for commitment to the *in situ* Hi-C workflow (Figure 1A). This experimental setup was well-suited to compare the fresh unstimulated *ex vivo* status with that induced by strong IL-2 receptor and IL-2 receptor + TCR + CD28 co-receptor signaling.

Isolated populations were >96.5% positive for cell surface expression of the established mCD4+ T-cell phenotypic markers (Figure 1B). A representative donor is shown in Figure 1C (see Supplementary Figure S2 for the other two donors) for each condition. As expected, the IL-2 and IL-2+TCR treatment increased the size of the cells, which is detected as a widening of the distribution of the treated relative to the unstimulated cells in the forward scatter channel (FSC). To measure the physiological effect of these treatments, we used the activation markers CD25 (IL2-Rα), a component of the heterotrimeric IL-2 cytokine receptor, and CD69 (cell adhesion molecule), an early activation marker (Simms and Ellis, 1996) (Figure 1C; Supplementary Figure S2). A proportion of unstimulated cells expressed low levels of CD25, consistent with its pro-survival function, but only ∼15% of the cells expressed both CD25 and CD69, indicating that they were mostly quiescent. The levels of CD25 surface expression and the proportion of CD25+ CD69+ cells increased upon treatment with IL-2 and reached >60% upon TCR + CD28 stimulation (Figure 1C; Supplementary Figure S2). These results were confirmed by comparing the expression strength level or mean fluorescent intensity (MFI) for CD69 and CD25 (Figure 1D; Supplementary Figure S2). Lastly, we assessed the cell cycle profile of the cells using propidium iodide and found that in the unstimulated population, most of the cells were in G1 (resting state), whereas IL-2 stimulated entry into the cell cycle, and this effect was clearly more pronounced in the IL2+TCR population (Figure 1E; Supplementary Figure S3). Taken together, these results demonstrate that we obtained, in a reproducible way, three phenotypic states in mCD4+ T cells.

### Gene expression analysis confirms the activated phenotype of mCD4+ T cells

**FIGURE 2:**
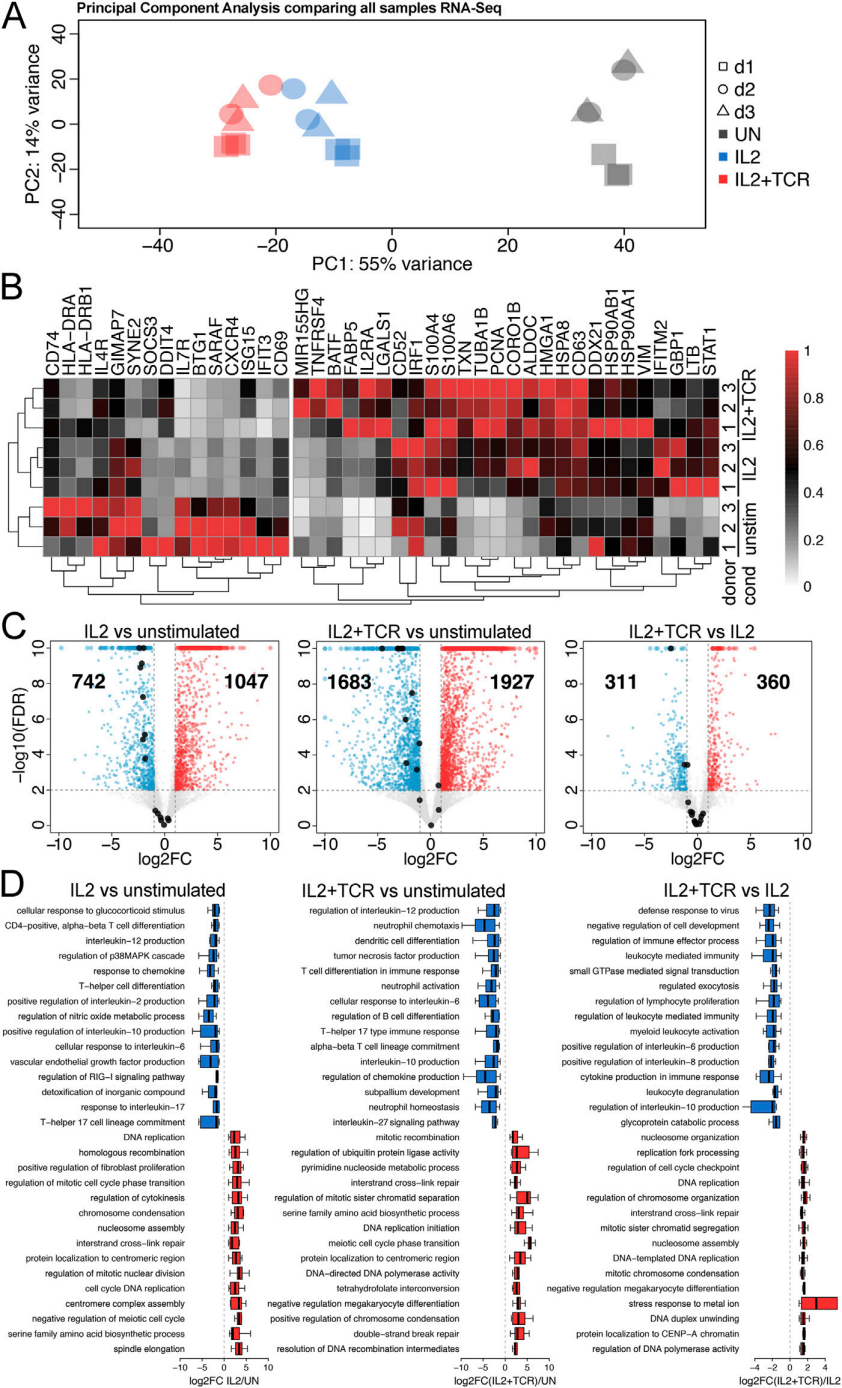
Gene expression changes between the activation conditions. **(A)** Principal component analysis (PCA) comparing two technical replicates for each of the three conditions (unstimulated, gray; IL2 alone, blue; IL2 + TCR activation, red) for each of the three blood donors (donor 1, squares; donor 2, circles; donor 3, triangles). **(B)** Heat map comparing donors 1, 2, and 3 across the three conditions. Clustering matches a previous study (Robinson et al., 2011), showing that activation is robust and follows the expectations of the literature. A 15-gene cluster of genes preferentially expressed in the unstimulated cells is indicated on the left. **(C)** Volcano plots comparing RNA-seq data for IL2 vs. unstimulated, IL2 + TCR activation vs. unstimulated, and IL2 alone vs. IL2 + TCR activation. Upregulated genes are denoted in red, and downregulated genes are denoted in blue. The cluster of 15 genes from **(B)** is highlighted with black dots. **(D)** Top GO Biological Process terms linked to upregulated (red) and downregulated (blue) genes are shown.

To examine the gene expression profile of unstimulated, IL2, and IL2+TCR mCD4+ T cells, we extracted RNA at days 0 and 5 and performed RNA-seq at ∼44 million paired-end (PE) reads per sample in x3 (d1) or x2 (d2 and d3) technical replicates for each condition and donor (Supplementary Table S1). PCA showed greater similarity across donors than experimental conditions and clear separation between each condition (Figure 2A). We performed differential gene expression analysis to compare conditions (Supplementary Table S3). We examined a set of 41 genes previously reported to define a signature of TCR-activated mCD4+ T cells in Soskic et al. (2022). We found that a cluster of 15 genes established as higher in transcript abundance in resting T cells was downregulated in the IL2 and IL2+TCR samples, two of them stepwise (*GIMAP7*, *SYNE2*) (Figure 2B). The panel of 26 genes upregulated on resting to effector T-cell fate change increased in either or both the IL2 or IL2+TCR samples (Figure 2B). Globally, for donor 1, IL-2 significantly upregulated 1,047 genes and downregulated 742 genes compared to unstimulated cells (Figure 2C). TCR stimulation substantially increased the differentially expressed genes over that of the IL-2 alone, with 1,927 upregulated and 1,683 downregulated genes. To better understand the stepwise changes that occur during T-cell activation and distinguish priming by IL-2 from full activation, we also compared the IL2 and IL2+TCR-stimulated cells, revealing 360 upregulated genes and 311 downregulated genes (Figure 2C), indicating high overlap. Donor 2 and 3 transcriptome changes matched those of donor 1 (Supplementary Table S4).

Next, we used g:Profiler to identify functional GO-terms associated with the induced/repressed genes. To plot associated GO-terms in an unbiased manner, we first sorted gprofiler outputs according to enrichment for a particular term over the expected and plotted the 15 highest enriched terms. Because there is considerable redundancy between the GO-terms themselves, with often different terms for the same gene set and new terms assigned with only slight changes in the composition of a gene set, we further removed redundant terms. The full lists of GO-terms are included in Supplementary Table S2. This analysis (Figure 2D) indicated that for both the IL2 and the IL2+TCR samples compared to the unstimulated, the most upregulated pathways were associated with cell cycle activation and cell proliferation (e.g., DNA replication, chromosome segregation, and histone production) (Figure 2D, red bars), in agreement with the observed cell proliferation profile (Figure 1E; Supplementary Figure S3). Additionally, several terms associated with DNA damage could reflect the need to upregulate this pathway to maintain genome integrity during bursts of rapid cell division. One metabolic pathway (serine family amino-acid biosynthetic process) made the top 15 list for the IL2 versus unstimulated condition, and this function was further enhanced with full activation in the IL2+TCR versus unstimulated condition.

The most enriched downregulated GO-functional terms (Figure 2D, blue bars) in both IL2 and IL2 + TCR samples mainly defined pro-inflammatory pathways. These included IL-6, IL-12, IL-17, and tumor necrosis factor-alpha (TNFα) (54, 55), although we also observed downregulation of the IL-10 pathway, which is anti-inflammatory (Frucht, 2002). Similar results observed for all three donors were analyzed separately (Supplementary Figure S5). These results indicated that the stimulated cells had a late effector phenotype and were less pro-inflammatory, with a reduced Th17-like transcriptional signature.

### Robustness and reproducibility of *in situ* Hi-C on mCD4+ T cells using the commercial *in situ* Hi-C (Arima) kit

**FIGURE 3:**
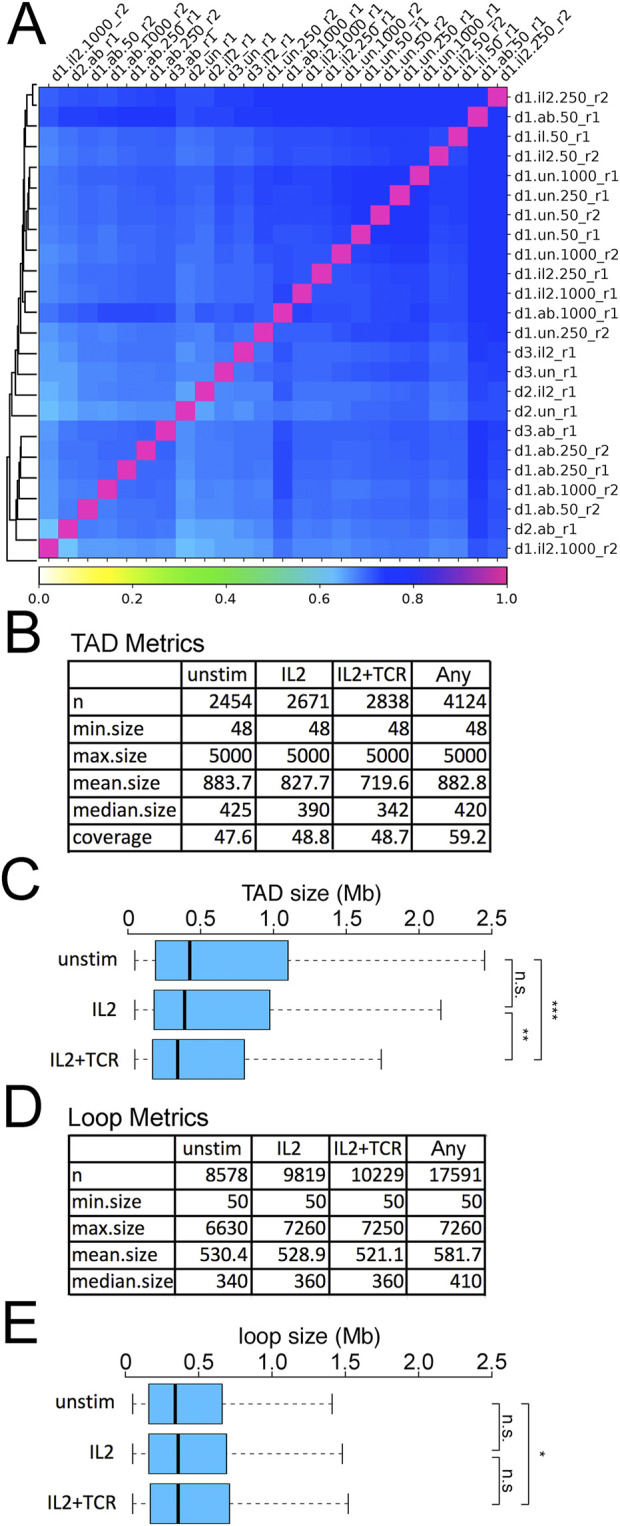
Reproducibility of Hi-C data and metrics. **(A)** Correlation matrix for UN, IL2, and IL2+TCR samples for donors 1, 2, and 3 and their replicates. The column and row names identify the specific samples using a 4-term string, with a dot or underscore as a separator. The first term (d1, d2, or d3) indicates the donor. The second term indicates the treatment (un = unstimulated, il2 = IL2 treatment, ab = IL2+TCR treatment). The third term indicates the number of cells harvested (50 × 10^6^, 250 × 10^6^, or 1,000 × 10^6^), and the last term indicates the replicate (r1 or r2). **(B)** TAD metrics for numbers, size, and genome coverage are listed for the different conditions in donor 1. The label “any” corresponds to the non-redundant set of TADs detected in at least one of the conditions. **(C)** Plotting the distribution of TAD sizes between the three conditions revealed a decrease in TAD size during activation. The boxplot whiskers extend to 1.5× the interquartile range, with outliers omitted. **(D)** Loop metrics for numbers and sizes. The label “any” corresponds to the non-redundant set of TADs detected in at least one of the conditions. **(E)** Plotting the distribution of loop sizes between the three conditions revealed a slight increase during activation. The boxplot whiskers extend to 1.5× the interquartile range, with outliers omitted. Kolmogorov–Smirnov test, *p < 0.05, **p < 0.01, and ***p < 0.001.

We next performed Hi-C on these cell populations. Because the number of mCD4+ T cells available from clinical samples is often limited, we tested 50 k, 250 k, and 1 M cells for each donor and condition. All conditions yielded similar quality final libraries (Supplementary Figure S6). To determine the global similarity of detected DNA-DNA contacts between conditions, we calculated the pairwise Pearson correlations between samples with Hi-C Explorer (Love et al., 2014) on the 10 kb resolution matrices (Figure 3A). This showed a good correlation between all samples (Pearson r values ranging between 0.65 and 0.8), indicating both high uniformity across donors and good clustering of the different conditions. The high degree of similarity suggests that there is an overall shared genomic 3D structure that only changes in minimal, albeit uniform, ways between experimental conditions/activation states (Figure 3A). There was remarkable consistency across the 50 k, 250 k, and 1 M samples despite different sequencing depths (Figure 3A; Supplementary Table S4).

TADs were called by HOMER (Bolger et al., 2014). Identified TADs had similar genomic coverage across all conditions, ∼48%, and were reasonably similar in size range; however, there was a moderate but steady increase in numbers of detected TADs and a decrease in mean size going from unstimulated to IL2 samples and again to the IL2+TCR samples (Figure 3B) (Quon et al., 2023). The overall reduction in TAD size was statistically significant (Figure 3C) and indicates increased intra-TAD contacts to the exclusion of contacts between TADs. Loops were called using an implementation of the HICCUP algorithm in Hi-C Explorer (Love et al., 2014). Although it is sometimes thought to be a general correspondence between loops and TADS (Hansen et al., 2018), roughly four times more loops were identified than TADs, and the literature indicates considerable variability depending on which algorithms are used. The number of loops also progressively increased from unstimulated to IL2+TCR samples (Figure 3D); however, their mean size became larger, showing an opposite trend relative to TAD size (Figure 3E). Although there was no difference in mean loop size between the IL2 and IL2+TCR samples, the IL2+TCR sample loops were marginally but significantly larger than in the unstimulated sample.

### Compartment switching occurs at different mCD4+ T-cell activation status

**FIGURE 4:**
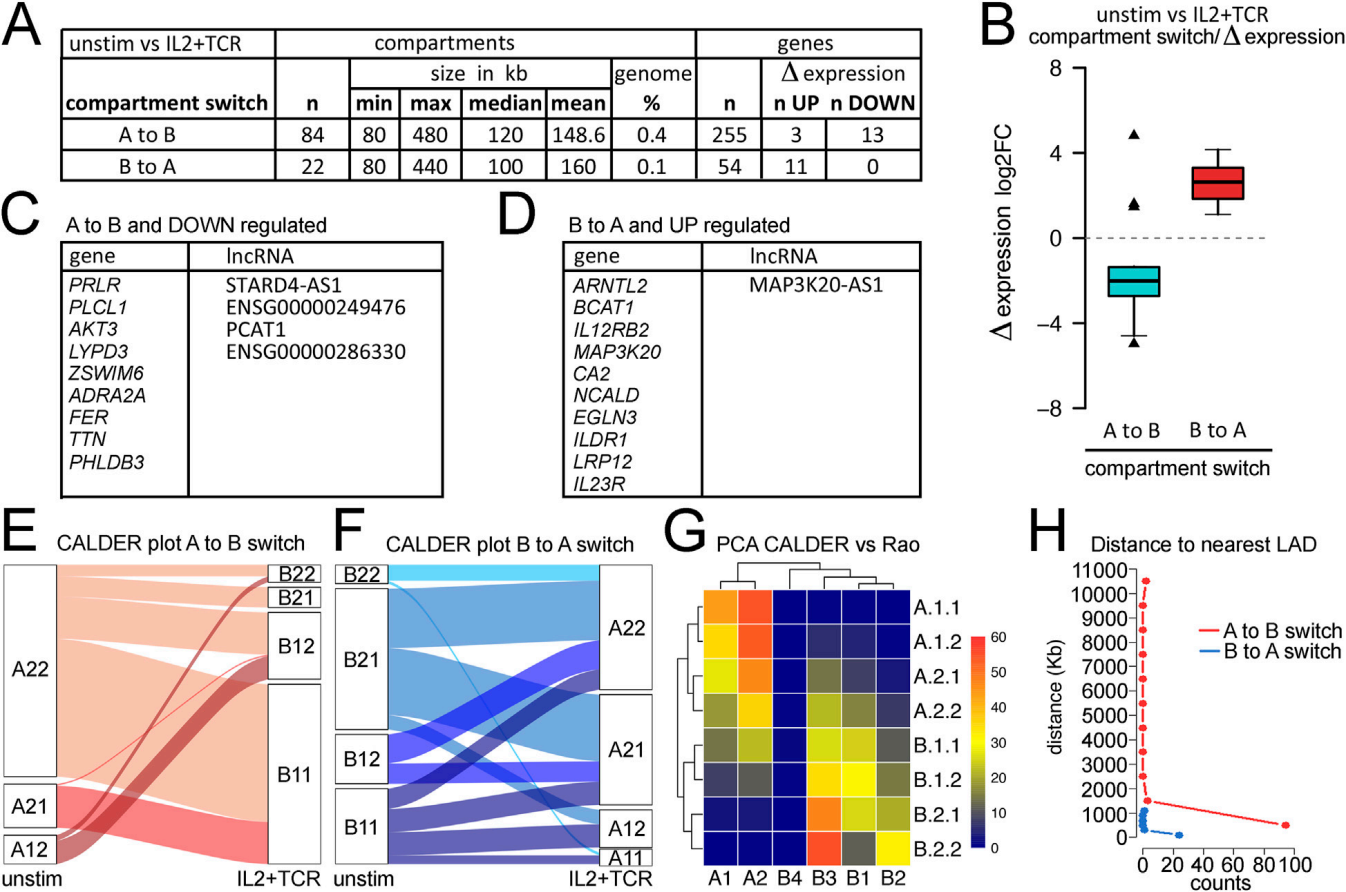
Analysis of compartments and compartment switching during mCD4+ T-cell activation. **(A)** Metrics for numbers and sizes of compartments switching from A to B or B to **(A)**. **(B)** Plot of compartment switches against gene expression changes for genes within the changing compartment. Nearly all A to B switches correlated with loss of expression, while all B to A switches correlated with a gain in expression. The enrichment of upregulated genes in the B to A switches or downregulated genes in the A to B switches was highly significant when compared to the overall proportion of up/downregulated genes (Fisher’s exact test, p = 0.0012 and 0.0096, respectively). **(C)** List of genes and lncRNAs in compartments switching from A to B while being downregulated. **(D)** List of genes and lncRNAs in compartments switching from B to A while being upregulated. **(E)** Breaking down the larger A and B compartments into CALDER-defined sub-compartments revealed principal switches from A.2.1. to B.1.1 and from A.1.2 to B.1.2. **(F)** In the opposite direction, CALDER-defined B.1.1 subcompartments changed to all A sub-compartments, while the B.1.2 sub-compartment only changed to A.2.1 and A.2.2 compartments. **(G)** Correspondence between CALDER-defined sub-compartments and Rao-defined (Serizay et al., 2024) A.1–2 and B.1–4 subcompartments for T cells derived with SNIPER (Xiong and Ma, 2019). **(H)** Plotting distance from a region switching compartments to nuclear envelope based on the nearest LAD using Jurkat T-cell activation data to define LADs.

The global decrease in TAD sizes and increase in the number of loops suggested a possible net increase in regulatory interactions. Another metric that can indicate 3D genome changes associated with gene activation or repression is A→B, or B→A, compartment switches (Supplementary Table S5). Using the general compartment calling to compare the unstimulated mCD4+ T cells with the IL2+TCR-stimulated cells revealed 84 genome regions moving from the A to B compartment and 22 regions moving from the B to A compartment, and the size of the moving compartments was similar in either direction (Figure 4A). These changes in compartment call represented only 0.4% and 0.1%, respectively, of the linear genome coverage and, therefore, could be important functional 3D switches modulating transcript output. The small coverage of these switches is similar to that found in naïve CD4+ T-cell activation (Quon et al., 2023). Most genes in these compartments did not change in expression. However, for those genes that did, the general tendency was for genes moving from the A to B compartment to become repressed and for those moving from the B to A compartment to become activated, except for three genes moving from the A to B compartment that were activated (Figure 4B). Downregulated genes did not appear to be relevant for CD4+ T-cell function, whereas upregulated genes included IL-12 receptor component *IL-12RB2* and the IL-23 receptor *IL23R*, which upon IL-23 ligation, induces proliferation of mCD4+ T cells (Frucht, 2002) and their polarization toward the Th17 type, and could thus promote the differentiation of the Treg1/17 sub-lineage (Hollbacher et al., 2020) (Figure 4C).

Several lncRNAs were among the transcripts that changed in expression, which can often function in the regulation of multiple genes. Although not much is currently known regarding the specific functions of most of these lncRNAs, MAP3K20-AS1, STARD4-AS1 and PCAT1 are high-risk factors for different cancers (Levick and Knight, 1987; Mo et al., 2017; Ghafouri-Fard et al., 2020) and PCAT1 also is known to activate *SOX2* as well as affect cGAS/STING signaling (Gao et al., 2022).

Several studies used different approaches to refine compartment classification and identify multiple subcompartments. Serizay et al. (2024) inferred 2A and 4B subcompartments using a Gaussian hidden Markov model; however, this method requires very high sequencing depths, to the tune of several billion valid DNA-DNA contacts. The CALDER approach is a computationally lighter method that uses a divisive hierarchical clustering algorithm to segment the genome into regions according to their intra-to-inter-region similarity in terms of intrachromosomal interactions (Patro et al., 2017). CALDER was applied here to identify four A and four B subcompartments. We found that the frequencies of the subcompartments that switched varied. The A.2.2 sub-compartment more frequently moved to B.1.1 and B.1.2, whereas the A.2.1 compartment more frequently switched to B1.1. (Figure 4E). In the other direction, the B.1.1 and B.1.2 subcompartments more frequently moved to A.2.2 and A.2.1 (Figure 4F). An alternative approach presented by Rao et al. (2014) identified two A subcompartments and four B subcompartments. We previously observed that the Rao et al. B3 sub-compartment was mostly at the nuclear envelope in LADs, and many genes activated in Jurkat cells moved from the B3 sub-compartment to the A2 sub-compartment concomitant upon activation (Robson et al., 2017). Therefore, we compared the CALDER subcompartments to the Rao et al. subcompartments and found that the B3 sub-compartment most strongly corresponds to the CALDER B.2.2 sub-compartment but also included much of the B.2.1 and part of the B.1.1 and B.1.2 subcompartments (Figure 4G). Likewise, the Rao A2 sub-compartment most strongly corresponds to the A.1.1 and A.1.2 CALDER subcompartments (Figure 4G).

The partial overlaps between the Rao-defined B3/A2 and similarly exchanging CALDER-defined subcompartments suggested a potential role for the nuclear envelope in regulating these genome architecture changes. This is consistent with much literature showing a tendency for a significant proportion of heterochromatin to be at the nuclear envelope (Van de Vosse et al., 2011) as well as our previous finding that many important immune genes that are activated during lymphocyte stimulation are released from the Rao-defined B3 compartments at the nuclear envelope into the Rao-defined A2 sub-compartment where they remain close to the nuclear envelope (Robson et al., 2017). However, because the overlap was only partial, it was possible that pericentric heterochromatin, which has been shown to contribute to the regulation of several important adaptive immune genes that similarly switch compartments during development (Skok et al., 2001; Goldmit et al., 2005), was also contributing. Therefore, we plotted the linear genomic distance of each CALDER sub-compartment to the nearest LADs based on those previously defined in Jurkat cells (Robson et al., 2017). B.2.2 and B.2.1 were clearly the closest to the nuclear envelope (Supplementary Figure S7), consistent with these showing the greatest similarity to the Rao-defined B3 sub-compartment (Figure 4G). Although error bars were high for the other individual subcompartments, A.1.1 and A.1.2 were the closest to the nuclear envelope, consistent with these showing the greatest similarity to the Rao-defined A2 sub-compartment (Figure 4G). We separately plotted the distance to the nearest LAD for all genes in regions undergoing A to B switching and all genes in regions undergoing B to A switching based on the CALDER-defined compartments. This revealed that genes moving into B compartments could come from anywhere in the genome, while those moving from B to A compartments were all within ∼1,000 kb from the nearest LAD, and the vast majority were within 100 kb (Figure 4H). This strongly suggests that most genes moving from B to A compartments are released from the nuclear envelope. This, combined with similar findings from studies in different cell lines (Rao et al., 2014), could represent a key principle of spatial control of transcription output. Genes annotated with a threshold distance of a LAD border can be toggled in their expression by increased or decreased tethering to the nuclear envelope, in which the repressive chromatin state is typically found. This mechanism appears to be active in mCD4+ T cells, as shown here, representing a differentiated adult cell type.

### Loops and TADs undergo stepwise changes during mCD4+ T-cell activation

**FIGURE 5:**
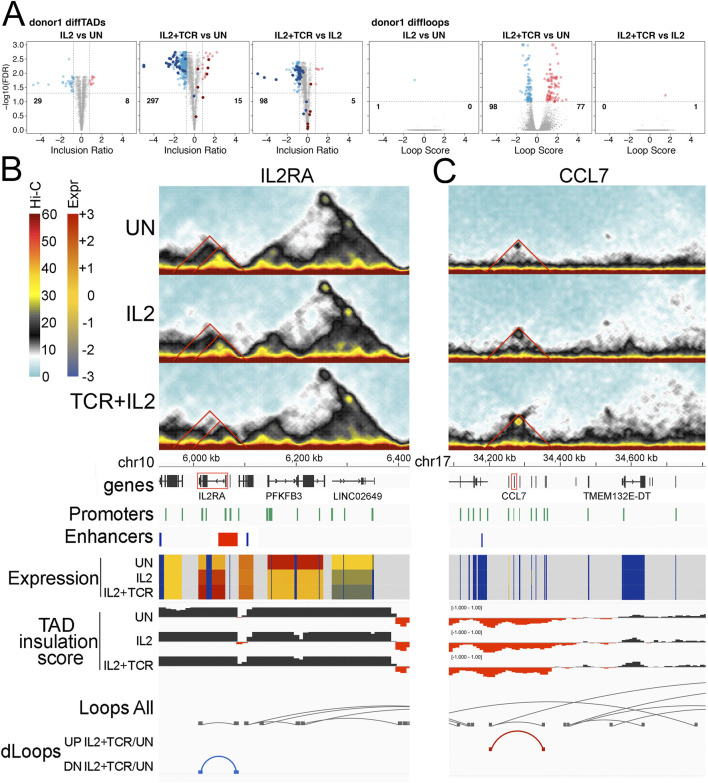
Differential TADs and loops. **(A)** Volcano plots showing TAD (three left panels) and loop (three right panels) changes when comparing UN vs. IL2, UN vs. IL2+TCR, and IL2 vs. IL2+TCR conditions. A differential TAD or loop means that the TAD inclusion scores or the loop interaction frequencies change significantly. This could indicate gain/loss, a significant change in boundaries, or a significant change in the number of interactions within a TAD. In all cases, red indicates an increase, while blue indicates a decrease. Differential TADs between unstimulated and IL2 treatment (first panel) were highlighted in the comparisons unstimulated against IL2+TCR and IL2 against IL2+TCR (panels 2 and 3) using dark red and dark blue for up- and downregulated, respectively. **(B–C)** Hi-C interaction maps for particular TADs are given on the top three panels for the status in each condition, with UN on top, IL2 in the middle, and IL2+TCR on the bottom. The genomic tracks underneath indicate, respectively, chromosome coordinates, Ensembl Hg38 v.106 genes (black), promoter positions (green), Enhancer Atlas CD4+ T-cell enhancers (blue), gene expression indicated as zFPKM (z-score transformation of fragments per kilobase per million pair-end reads, FPKM), where low values (blue) indicate no or very low expression and high values (red) indicate high expression levels, TAD insulation scores that dip at TAD boundaries, all loops detected regardless of condition (gray), and differential loops—UP or down (DN) for comparing only the IL2+TCR to the UN condition. **(B)** *IL2RA* locus. **(C)** *CCL2-13* locus.

To further dissect the impact of IL-2 and IL-2+TCR + CD28 stimulation on the spatial genome organization of mCD4+ T cells, we looked at TADs and loops that changed significantly in their interaction patterns between treatments using HOMER (Supplementary Tables S6, S7). A strong tendency was observed for TADs to undergo losses during mCD4+ T-cell activation, with roughly 20× more TADs lost or diminished than gained or increased in size between the UN pool and the IL2+TCR pool (Figure 5A, left panels). Note that these differential TAD changes more commonly reflected either significant changes in TAD boundaries or significant increases/decreases in interactions detected within a TAD than outright gain/loss of an entire TAD. Although roughly half of these changes required the combination of the IL-2 treatment and TCR + CD28 stimulation, a comparison of the UN and IL2 pools showed that IL-2 treatment alone could generate roughly a tenth of the overall changes and comparison of the IL2 and IL2+TCR conditions could account for roughly a third of the overall changes. Even though roughly four times more loops were detected than TADs (Figure 3D), there were comparatively few differential (gained or lost) loops, with nearly all changes induced only by IL2+TCR treatment. However, unlike the differential TADs, the numbers of gained and lost loops were similar, and the total numbers of differential loops were only about half the number of differential TADs. The addition of IL-2 alone changed TADs without corresponding changes in loops, whereas in the IL2+TCR samples, gains of loops were not accompanied by gains in TADs (Figure 5A, right panels).

To assess in greater detail the functional implications of these changes in TAD and loop organization, we examined two relevant immunological genes, *IL2RA*, whose expression was upregulated upon stimulation, and *CCL7*, whose expression was low and stable. The *IL2RA* locus appears to be part of a TAD that includes a sub-TAD and a loop (Figure 5B top Hi-C traces and loop tracks). This region includes several ENCODE annotated promoters and borders with an enhancer at the 5′ end (Figure 5B). Whereas the TAD, sub-TAD, and the loop diminish upon stimulation, a smaller region gains contact strength in correspondence with the 5′ region of *IL2RA* (Figure 5B middle and lower Hi-C traces and loop tracks). Notably, a STAT-5-dependent super-enhancer is located at that position (Vella et al., 1998; Van de Vosse et al., 2011; Skok et al., 2001). This suggests a progressive weakening of the original enhancer-promoter interactions found in unstimulated cells, which are replaced by the STAT-5 super enhancer-promoter interactions upon stimulation. Here, we capture the SE wide contact increase at the expense of the longer-range interactions in the unstimulated landscape. This concentration of STAT5 binding motifs presumably occurs to boost expression in response to IL-2 signaling as part of the feedback circuitry.

Another interesting gene cluster is that containing *CCL2*, *CCL7*, *CCL11*, *CCL8*, and *CCL13* (Figure 5C, *CCL7* is highlighted in red). The set of secreted ligands (chemokines) encoded at this locus are important for recruiting immune cells through the ligation of G-protein coupled chemokine receptors that coordinate changes in the migratory cytoskeleton (Goldmit et al., 2005). There is a faint TAD structure in this region in the unstimulated cells, which includes several ENCODE annotated promoters and an enhancer at the outer 5′ boundary (Figure 5C top Hi-C traces). Progressively after IL-2 stimulation and then IL-2+ TCR + CD28 stimulation, this TAD fills in and becomes more solid, and a new loop forms that defines its edges (Figure 5C, middle, and bottom Hi-C traces and loops tracks). These structural changes did not correlate with changes in expression, which remained low (Figure 5C, Expr tracks). We suggest that the 3D shifts at these loci could be priming or de-priming the loci in advance of further TF mobilization not induced under these *ex vivo* culture conditions.

### Changes in enhancer–promoter interactions occur in each step of stepwise activation but often do not correlate with expression changes

**FIGURE 6:**
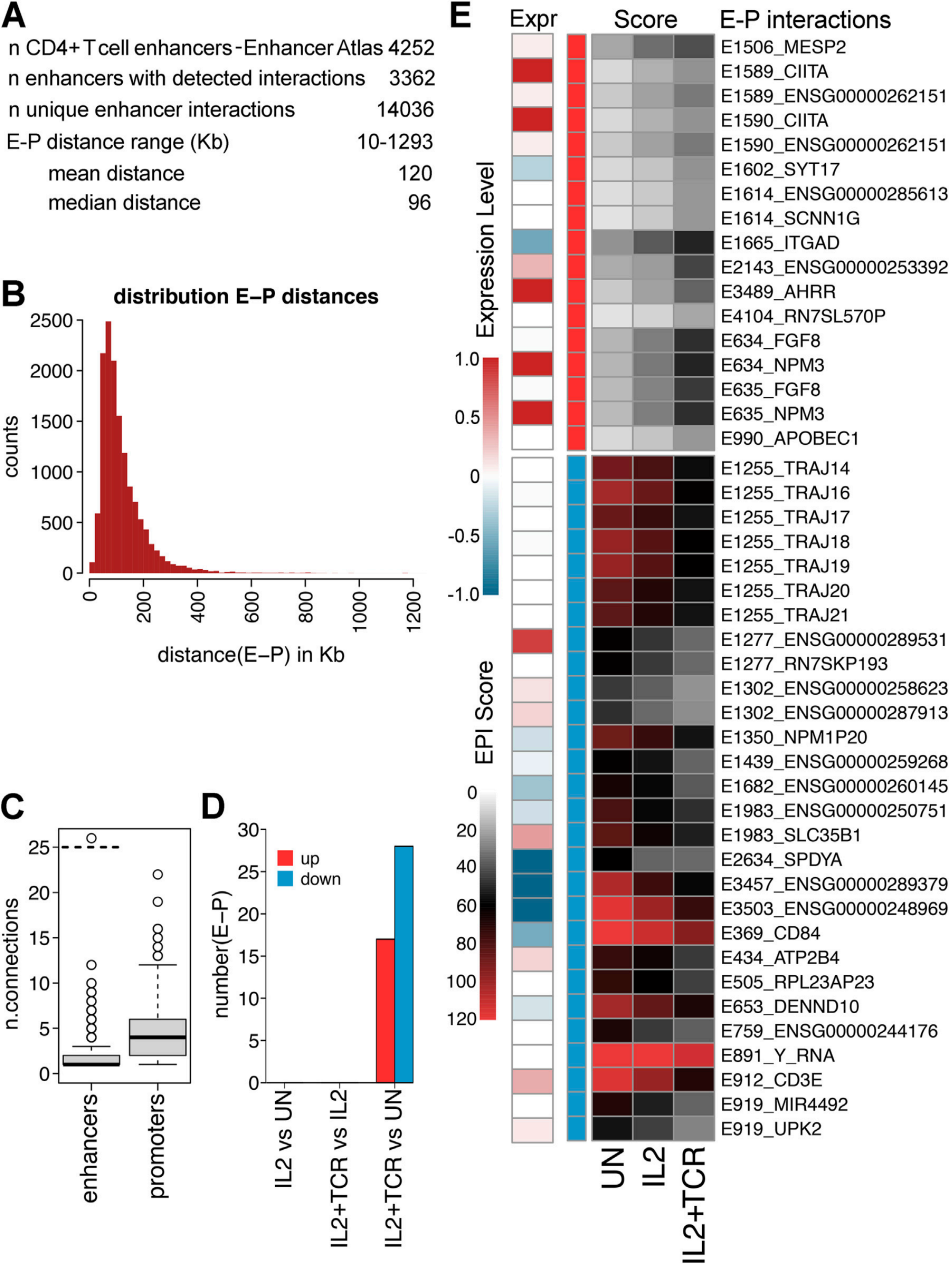
Differential enhancer–promoter interactions during stages of activation. **(A)** Metrics for enhancers and promoters and their interaction. Enhancer–promoter pairs from unstimulated IL2 and IL2+TCR treatment in donor 1 were pooled. **(B)** Histogram plot of the distribution of enhancer–promoter (E–P) distances. Enhancer–promoter pairs from unstimulated, IL2, and IL2+TCR treatment in donor 1 were pooled. **(C)** Box plot of numbers of connections between each enhancer and promoters or *vice versa*. **(D)** Numbers of enhancer–promoter (E-P) interactions identified in each comparative condition. **(E)** Heatmap of interaction scores for 17 increasing and 28 decreasing (top red and bottom blue, respectively) E-P interactions that changed with statistical significance (FDR 5%) between UN and IL2+TCR. Changes appear to be stepwise, with the IL2 treatment being intermediate between UN and IL2+TCR. Expression changes—log2FC (IL2+TCR/UN)—are shown under “Expr.”

To systematically explore how stimulation may change enhancer-promoter interactions in mCD4+ T cells, we used the set of reported enhancers for human CD4+ T cells from Enhancer Atlas 2.0 (Heinz et al., 2010) to query our Hi-C data using a virtual 4C approach in order to identify enhancer-promotor interactions (EPIs). Altogether, there were 4,252 defined enhancers, and we detected interactions for roughly three-quarters of them (3,362) (Figure 6A). The distance range on the linear genome between enhancers and promoters ranged from 10 kb to over a Mb with a peak at approximately 100 Kb (Figure 6B). The greater tendency was for an enhancer to have multiple interactions such that these 3,362 enhancers had a total of 14,036 EPIs (Supplementary Table S8). Nonetheless, most enhancers only interacted with a few (2–3) promoters, though one (Y_RNA) interacted with 168 enhancers (Figure 6C). Promoters, on the other hand, had a tendency to interact with more enhancers (on average ∼5; Figure 6C). Enhancers are often thought to interact with multiple co-regulated genes. Indeed, in many cases, an enhancer interacted with multiple immune genes, though more often, there was a mixture of immune and other types of genes for those enhancers with the most EPIs, suggesting a competition between enhancers and the promotors of genes with differing functional annotations for positioning within the connectome.

Despite the large number of measured EPIs, there were only a small number of differential EPIs (i.e., ones that changed between the different conditions), and these all occurred only with the combined IL-2 and TCR activation treatment (Figure 6D). EPIs are known to increase the level of expression at a locus (Zhou, 2016). It is generally thought that EPI establishment occurs at loci already undergoing expression and may even be driven in part by this initial expression (Martin et al., 2001). Although the scores for EPIs only reached our statistical cutoff with the full activation, there was a clear trend for these differential EPI changes to be established after IL-2 stimulation, whether they were increasing or decreasing (Figure 6E). Notably, the same enhancer often exhibited similar interaction changes with multiple genes. Although the general tendency was for higher or lower EPI scores to correlate with respectively increased or decreased expression or no changes, the inverse occurred in 10%–20% of cases (Figure 6E). This could reflect an incomplete identification of all enhancers, as in the case of the IL2RA super-enhancer (Figure 5B), or differences in the mobilization of TFs.

*CIITA* and *AHRR* were among the loci with a progressive strengthening of both EPIs and gene expression. CIITA promotes elongation of the MHC-I and MHC-II gene transcripts (Pahl et al., 2024). Although functional MHC-II expression has not been established in T cells, MHC-I expression is increased on infection/inflammatory signaling to promote antigen presentation. *AHRR* encodes for the aryl hydrocarbon receptor (AHR) repressor, which inhibits AHR, a transcription factor that promotes mCD4+ T-cell polarization toward the pro-inflammatory Th17 lineage (Zhou, 2016). The *ITGAD* locus showed the opposite trend, with a higher EPI score but some reduction in expression. *ITGAD* encodes for CD11d, a β2 integrin important for leukocyte migration and extravasation (Yang et al., 2020). Its lower expression upon full stimulation may reflect a late effector cell profile, with a reduced migration and an enhanced tissue retention phenotype (Zhang et al., 2023).

Among the loci with reduced EPIs, *E1255* significantly changed its interactions with a set of *TRAJ* genes at the somatically recombined TCRα loci itself. This enhancer and its 3D interaction with the joining (J) gene promotors at this locus in the germline are responsible for genetic recombination in the thymus (Chin et al., 2022). Each T-cell clone will have selected a single J gene segment from the 51 functional options during thymic development, with those detected here being the most abundant. We suggest that the loss of promiscuous EPIs detected here echoes the past developmental interactions that generated the T-cell repertoire, highlighting the 3D contact changes are not always functional in a given context. The *CD84* locus showed progressive loss of EPI strength and downregulation of expression. CD84 is a co-stimulatory receptor that functions as both an immune activator and repressor dependent on inflammatory context (Martin et al., 2001).

**FIGURE 7:**
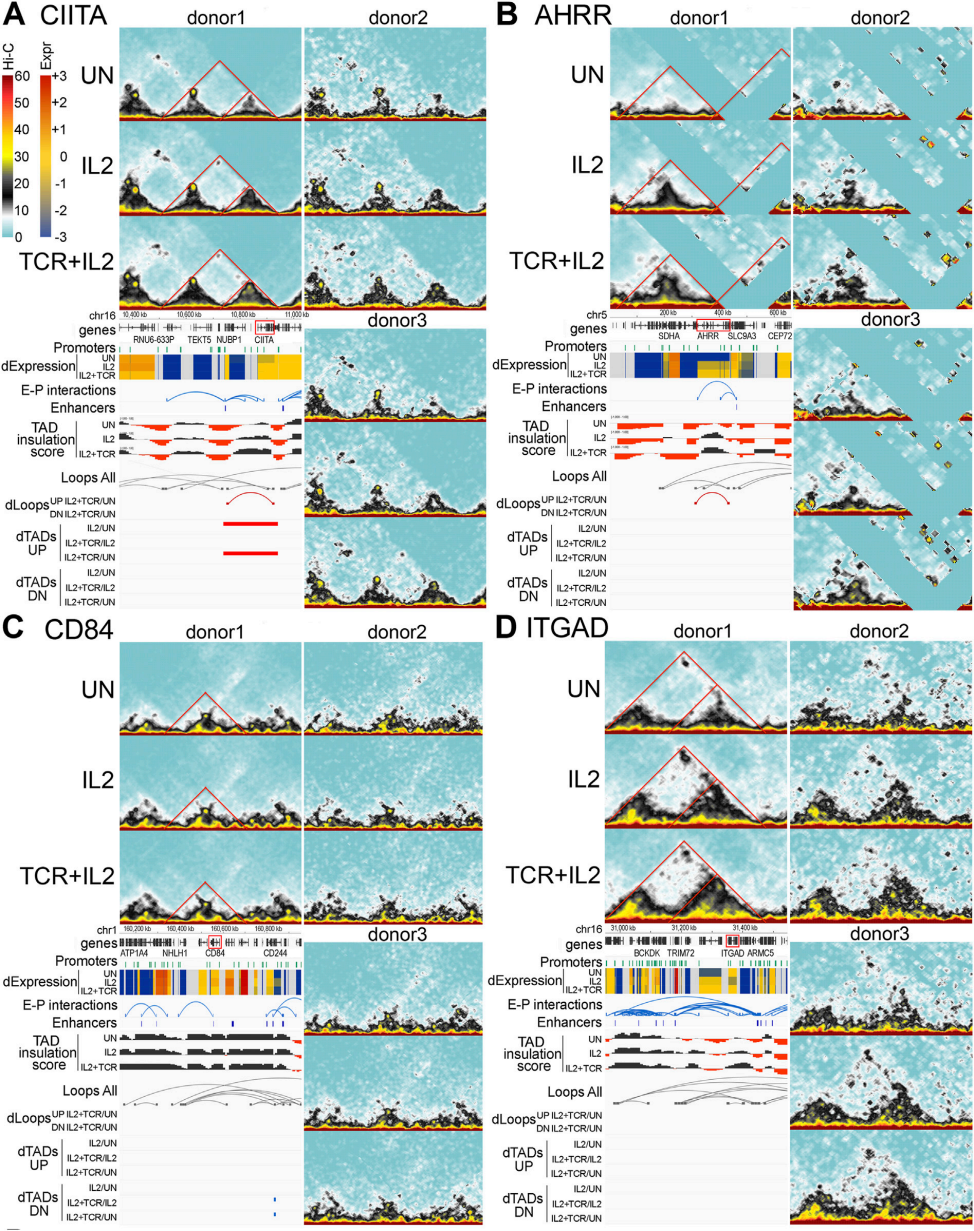
Four examples of important immunological genes with sequential changes in the TAD structure. **(A–D)** Hi-C interaction plots for each gene are shown from each donor for all three conditions (UN, IL2, and IL2+TCR). Annotation tracks are shown under donor 1 plots showing, from top to bottom, chromosome position, Ensembl Hg38 v.106 genes (black), promoters (green), and gene expression indicated as zFPKM (z-score transformation of fragments per kilobase per million pair-end reads, FPKM), where low values (blue) indicate no or very low expression and high values (red) indicate high expression levels, enhancer–promoter interactions (blue), Enhancer Atlas CD4+ T-cell enhancers (blue), TAD insulation scores which dip at TAD boundaries, all loops detected regardless of condition (gray), and differential loops up (UP, in red) or down (DN, in blue) for comparing only IL2+TCR to the UN condition and differential TADs both UP (in red) and down (DN, in blue) for all condition comparisons. **(A)** The *CIITA* locus. **(B)** *CD84* locus. **(C)** *ITGAD* locus. **(D)** *AHRR* locus.

To further investigate the spatial rearrangements of EPIs leading to changes in gene expression, we integrated the Hi-C traces with EPI tracks and TADs and loop changes (Figure 7). At the *CIITA* locus, a differential TAD became progressively larger upon IL-2 and IL-2+TCR + CD28 stimulation. As the TAD expanded, a loop also formed, connecting an enhancer located approximately 100 Kb upstream of the *CIITA* gene to its internal promoter (Figure 7A). These changes presumably strengthened EP contacts to induce higher gene expression. Notably, this same sequential set of 3D structural changes was observed in all three donors despite the much lower number of reads and sampling for donors 2 and 3 (Figure 7A, other donor Juicebox tracks).

The *AHRR* locus exhibited a TAD progressively developing upon IL-2 and IL-2+TCR + CD28 treatment (Figure 7D). A differential loop emerged upon cell stimulation, bringing an enhancer located at the 3′ end of the gene in proximity to the first 5′ *AHRR* promoter. These spatial changes were reproducible across the three donors and occurred in parallel with an upregulation of *AHRR* expression, supporting their functional relevance. Notably, for this region of the genome, there were several areas where reads could not be mapped either because they were highly repetitive or because this donor did not contain the number of repeats in the reference genome. Thus, *CIITA* and *AHRR* seemed to share a similar set of spatial changes upon stimulation, bringing enhancer and promoter into greater proximity, which presumably led to higher gene expression.

The spatial changes at the *CD84* and *ITGAD* loci were more subtle. For *CD84*, a loop located at the edge of a TAD appeared to become stronger in the IL-2-stimulated cells and weaker in the IL-2 + TCR-stimulated cells (Figure 7B). The loop included an enhancer located next to the CD84 gene and its promoter. As the loop strengthened in IL-2-treated cells, so did *CD84* gene expression, and when the loop decreased in the IL-2+TCR + CD28 stimulated cells, CD84 expression became lower, supporting a functional outcome of the loop changes. Although the changes were too subtle to reach our statistical threshold for differential loop calling, they were reproduced in all three donors.

For *ITGAD*, a large TAD encompassing two sub-TADs was detected. The sub-TAD closer to the *ITGAD* gene expanded and solidified. Moreover, at the apex of the main TAD, a loop was visible, which increased in IL-2-treated cells and then weakened in IL-2 + TCR-stimulated cells. This loop encompassed several enhancers that contacted the *ITGAD* promoters as well as multiple promoters of the *TRIM72* and *BCKDK* genes (Figure 7C), both encoding housekeeping-like metabolic functions. Because *ITGAD* gene expression decreased and increased in parallel with the changing loop size, we speculate that the loop might have shifted the balance of interactions disfavoring *ITGAD* promoter contacts with the enhancer at the *TRIM72* locus while favoring enhancer contacts with promoters of neighboring genes (i.e., BCKDK), which, contrary to *ITGAD*, showed stronger expression upon IL-2 stimulation relative to the unstimulated samples. Thus, the spatial changes at the *CD84* and *ITGAD* loci seemed to share a similar behavior, characterized by a loop becoming stronger in the IL2 samples relative to both untreated and IL2 + TCR samples, except that the functional outcome of such changes was the opposite.

## Discussion

We were able to generate high quality maps from 50,000 mCD4 T cells with two repeats and three conditions from the same blood donor using a commercially available (Arima) kit demonstrating that the large cell numbers ∼10,000,000 committed in many primary cell studies to date (Bediaga et al., 2021; Pahl et al., 2024; Johanson et al., 2018; Yang et al., 2020; Zhang et al., 2023) are unnecessary. Moreover, Hi-C maps were reproducible between three different donors. One caveat of this latter observation is that we started with a highly enriched subpopulation of CD4+ memory T cells, and it is possible that reproducibility correlates with such enrichment; however, we did not test this by comparing it to mixed populations. In either case, the data presented here should build confidence among the growing 3D chromatin architecture community that Hi-C contact maps can be routinely generated along with other functional genomics assays from rare or otherwise limiting primary material.

Although the combined stimulation with IL-2 and TCR activation has long been used as a standard for T-cell activation (Chin et al., 2022), to our knowledge, 3D genome-wide approaches (Hi-C) have not been widely used to test for distinctions between the effects of the IL-2 and TCR + CD28 activation. IL-2 is produced by activated CD4+ T cells and regulates key physiological responses, including CD4+ T-cell survival and proliferation, differentiation toward the Treg, Th1, and Th2 lineages, and suppression of the Th17 lineage (Kim et al., 2017). Furthermore, IL-2 is an emerging target to enhance the effectiveness of cancer immunotherapy (Spolski et al., 2018). Therefore, a better understanding of how IL-2 affects chromatin spatial conformation may lead to improved strategies to regulate its effects. Treatment with IL-2 alone caused many changes in TAD structure and EPIs. Although treatment with the combined IL-2 and TCR activation caused about ten times more changes, our testing of the IL-2 alone suggests a potential stepwise activation where each stimulus builds up the 3D chromatin structure. One enticing hypothesis for a function of such stepwise 3D genome organization changes is that IL-2 may promote the formation of TADs or loops that “prime” further strengthening and/or rearrangement of 3D structures upon TCR stimulation and additional TF mobilization. By manual inspection, we also found reproducible 3D chromatin changes specific to the IL2 samples, for example, at the *CD84* and *ITGAD* loci, that were reversed in the IL2 + TCR samples, pointing to specific functional effects driven by IL-2 signaling. These spatial changes had either positive, negative, or neutral effects on the expression of specific genes, underscoring the complexity of 1D and 3D drivers of gene expression.

The stepwise changes we found in the *IL2RA, CCL7, CIITA, CD84, ITGAD, AHRR,* and other loci may have functional similarity to the epigenetically imprinted, 3D priming described by Onrust-van Schoonhoven et al. (2023). In that study, differences in the 1D/3D epigenomic and transcriptomic circuitry were compared between naïve, memory, and effector Th2 cells. The resting memory Th2 genome maintained, throughout extended periods of homeostatic cell cycling *in vivo*, a profile of accessible chromatin and histone modifications in 1D as well as contact hubs in 3D that could be exploited more rapidly by TFs in the recall response. Whether these 3D structures were set up during naïve activation through the kind of stepwise buildup of the 3D genome structure shown here requires further investigation. This question is relevant for optimal vaccine design, where long-lived memory cells epigenetically primed for a specific inflammatory response are the primary goal (Wimmers et al., 2021). Our results also agree with previous studies (Wang and Bian, 2024; Abadie et al., 2024) that have investigated the contribution of shifts in the 3D genome structure in T cells, with the suggestion (Boltsis et al., 2021) that developmental differentiation is associated with clear loss/gain of 3D structures, while response to stimuli is associated with more subtle changes (Acemel and Lupianez, 2023).

We see the CD4+ T-cell subset as a system that merges the boundary between differentiating cell-fate transition and response to external stimuli. This plasticity has been selected for during evolution to ensure CD4+ T cells can modulate both the magnitude of their response to pathogenic challenges through the naïve vs. effector epigenomic circuitry and the type of response, whether inflammatory or immunosuppressive. The setup of the 3D genome during thymic development (Hu et al., 2018) allows for subtle and reversible 3D shifts in response to immune signaling to contribute to this plasticity. Of note, even with the 0.7B total contacts in the donor 1 Hi-C matrices presented here, most differentially expressed gene loci do not overlap with dynamic 3D contact calls, including EPIs. This suggests that the developmental set up of a well-buffered 3D connectome at many loci is exploited by TFs to increase expression in a way that does not require a population-wide change in chromatin conformation. Whether deeper sequencing (Harris et al., 2023) would reveal even more subtle 3D chromatin changes at these loci correlating with the relatively large expression changes (Rebeiz and Tsiantis, 2017) is unknown. Another caveat is that we used existing enhancer data from other studies of lymphocyte activation rather than enhancers specifically identified in our cell populations and thus might have missed novel enhancers that have yet to be identified specific to this cell type (Pahl et al., 2024).

3D compartment organization has been well-correlated to transcript abundance, histone modification patterns, and internal/peripheral nucleosome localization (Boltsis et al., 2021; Zheng et al., 2023), although it is unclear if the separation into A and B compartments is a cause (instructive mode) or an effect (reinforcing mode) of the differences in gene expression characterizing these compartments. Our study cannot address this issue. Nonetheless, the LAD compartment shifts correlating with expression changes that we detected provide support to previous work (Bediaga et al., 2021), suggesting that compartment-level dynamics are functionally relevant in differentiated mCD4+ T cells. In some cases, such as the *CCL7* locus, a TAD seemed to be formed to isolate genes from enhancers, presumably to shut down their expression. This might be an alternative repressive mechanism to the recruitment of genes to the lamina-associated B cell compartment (Robson et al., 2017). To reverse silencing, another signal causing de-condensation of the TAD could make the genes available again for interactions with enhancers outside the TAD.

The TAD/loop dynamic algorithmic calls are more difficult to reconcile exclusively with the initially hypothesized (Gonzalez-Sandoval and Gasser, 2016) function of the TAD to provide largely invariant insulation to functional loop/sub-TAD dynamics that influence EPI contact frequency. In the deeply sequenced donor 1 contact maps, we recorded greater dynamics in the TAD calls relative to the loop calls. On manual inspection, there is clearly a spectrum of 3D features from very punctate foci (*ITGAD* locus) of contacts that are definitively called as loops to clear blocks of contact neatly fitting within a called TAD (*CIITA* loci). Most gene loci (*CD84* as an example) include 3D contacts with both intense dots of contacts and most dispersed TAD-like contact signals. Attempts to algorithmically call this type of structure in the contact matrix have been reported elsewhere (Yoon et al., 2022). Our data suggest that instead of calling specific features in the contacts, an approach based on looking for increased/decreased contacts between promoters and the surrounding gene regulatory landscape, including sub-optimal enhancers (Lim et al., 2024), could be more productive in identifying functional contacts. The *IL2RA* locus indicates how challenging it will be to call these functional contacts vs the non-functional changes that are a consequence of these shifts in the 3D genomic structure or could represent the 3D equivalent of neutral genetic drift (Rebeiz and Tsiantis, 2017). The *CIITA* loci highlight an unexplored complexity in that this cell-type-specific developmentally regulated locus is highly interconnected with local housekeeping genes within the same TAD. How the regulation of these housekeeping gene promotors (Dejosez et al., 2023) and the differentiation-/stimuli-regulated *CIITA* loci are achieved is unknown.

In conclusion, we present here the technical details required to confidently commit populations of as few as 50,000 isolated T-cell subtypes to the established *in situ* Hi-C workflow using the commercially available Arima kit; we identify stepwise buildup of 3D chromatin structures upon IL-2 and IL-2 + TCR stimulation as a feature of mCD4+ T-cell activation, and we provide a publicly available data set to support future work investigating how 3D chromatin conformation supports T-cell function.

## Data Availability

The RNA-seq raw and processed data have been deposited with Gene Expression Omnibus (GEO) ID: GSE288509; https://www.ncbi.nlm.nih.gov/geo/query/acc.cgi?acc=GSE288509 and the Hi-C raw and processed data have been deposited with GEO ID: GSE288510; https://www.ncbi.nlm.nih.gov/geo/query/acc.cgi?acc=GSE288510.
