# Supplementary material for: Memory CD4+ T cells sequentially restructure their 3D genome during stepwise activation
Source: Front Cell Dev Biol. 2025 Feb 13;13:1514627. doi: 10.3389/fcell.2025.1514627 (PMC11866950; doi:10.3389/fcell.2025.1514627)
Supplement: Supplementary file 9 [file DataSheet1.docx]

Supplementary Material

# Supplementary Data

Supplementary Material should be uploaded separately on submission. Please include any supplementary data, figures and/or tables.

Supplementary material is not typeset so please ensure that all information is clearly presented, the appropriate caption is included in the file and not in the manuscript, and that the style conforms to the rest of the article.

# Supplementary Figures and Tables

For more information on Supplementary Material and for details on the different file types accepted, please see [here](https://www.frontiersin.org/guidelines/author-guidelines#supplementary-material).

## Supplementary Figures

Supplementary Figures 1-7


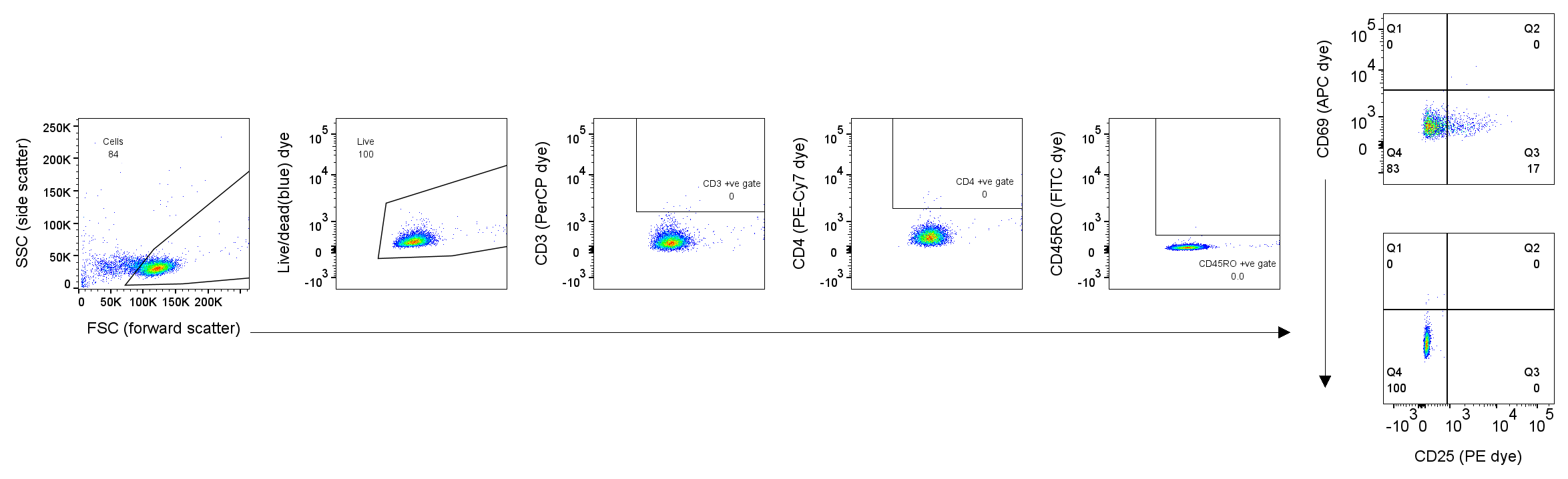


**Supplementary Figure S1.** Isolation of high purity primary memory CD4+ T cells by magnetic separation. Flow dot plots generated by staining an independent donor isolated mCD4 T cells with the 6-dye panel minus 1 dye. The placing of the +ve gates is shown for each dye.

**
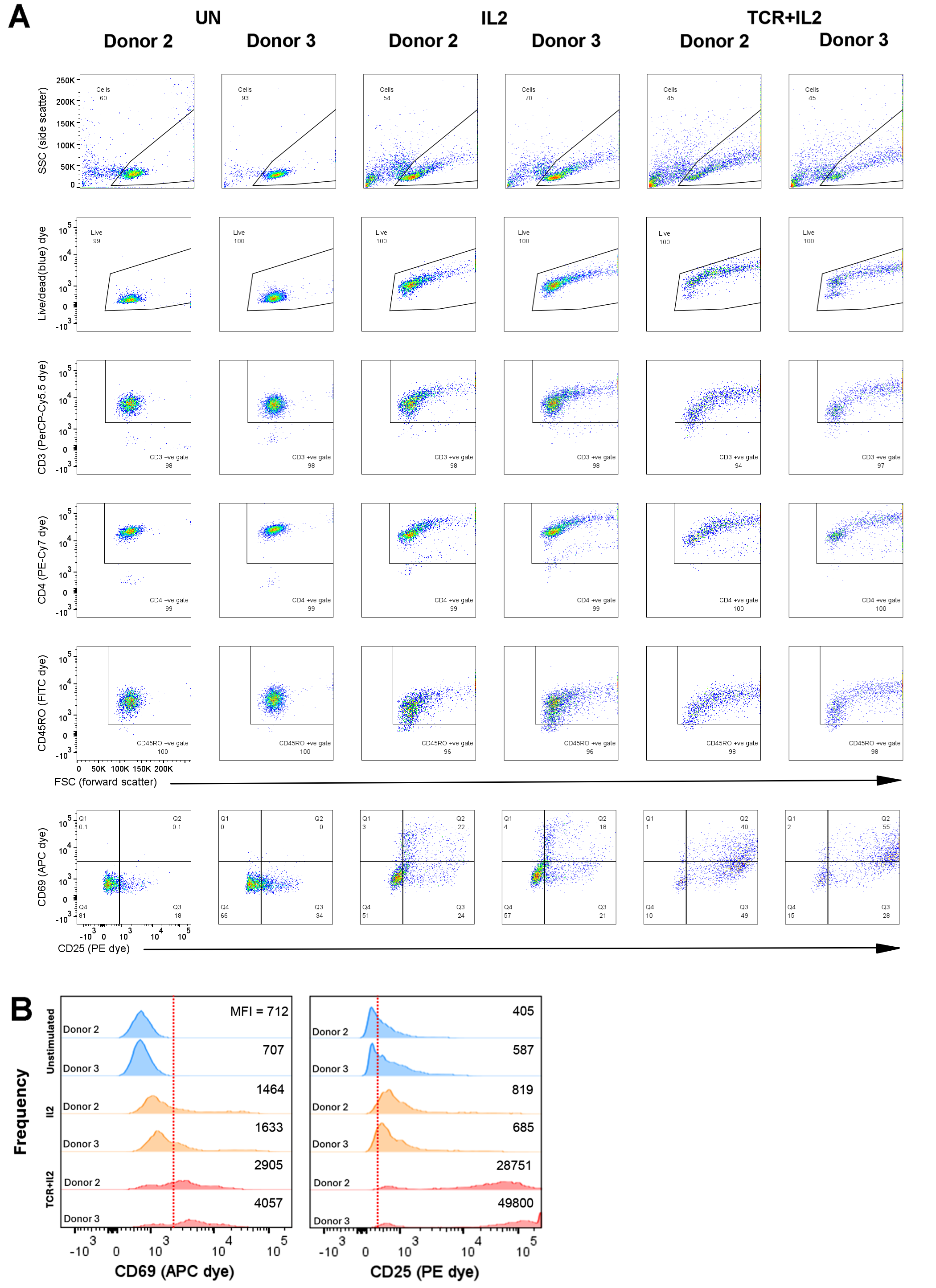
**

**Supplementary Figure S2.** Flow assessment of stimulated cell populations. **(A)** Flow dot plots for IL2+TCR stimulated donor 2 and 3 mCD4+ T cells and flow dot plots for IL2 stimulated donor 2 and 3 mCD4+ T cells. **(B)** Histograms showing the frequency of each bin of CD69/CD25 (APC/PE dye) expression for donors 2 and 3 as the unstimulated population and the IL2+TCR and IL2 alone stimulated populations. The vertical red line separates the CD69/CD25 -ve (left) from the +ve (right) population (MFI = median fluorescence intensity).

**
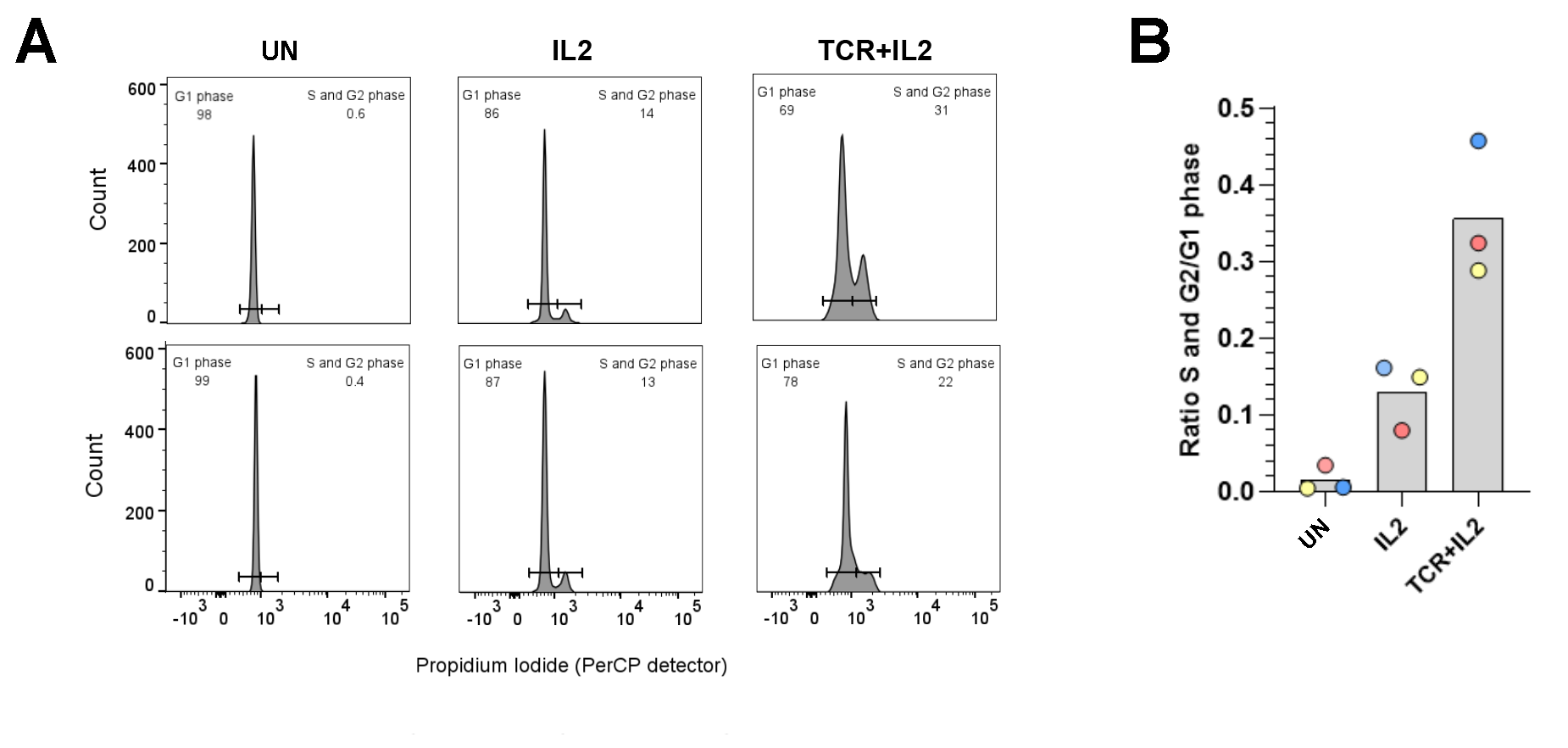
**

**Supplementary Figure S3.** Cell cycle phase assay. **(A)** Propidium Iodide staining for cell cycle phase assignment of donor 2 and 3 UN, IL2, and IL2+TCR stimulated populations, signal was captured using the PerCP detector. **(B)** Bar chart showing ratio of S and G2 phase/G1 phase flow dot plot events.

**
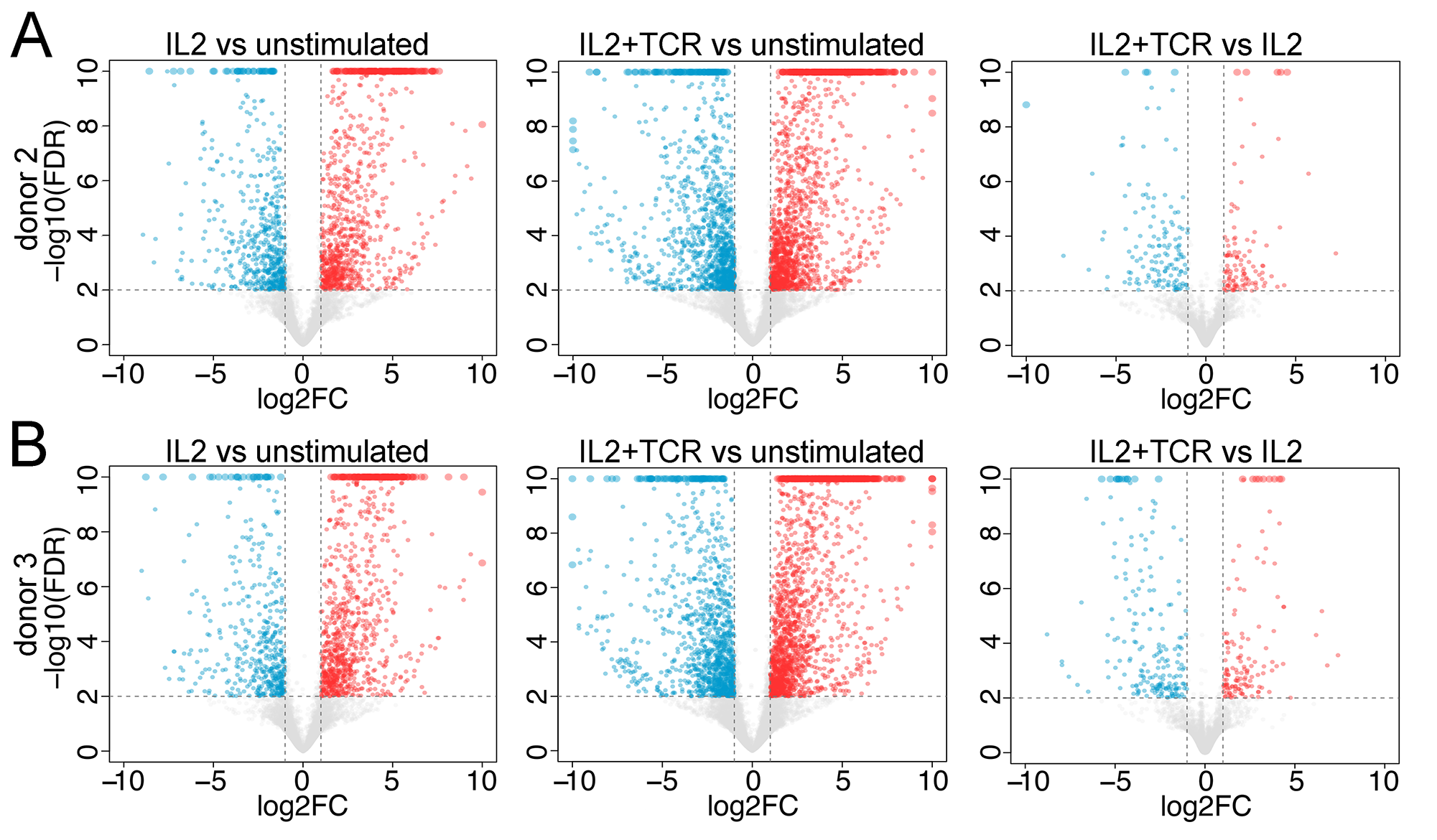
**

**Supplementary Figure S4.** Differential gene expression volcano plots for donors 2 and 3 matched to donor 1 data shown in Figure 2, panel C. **(A)** Donor 2. **(B)** Donor 3.

**
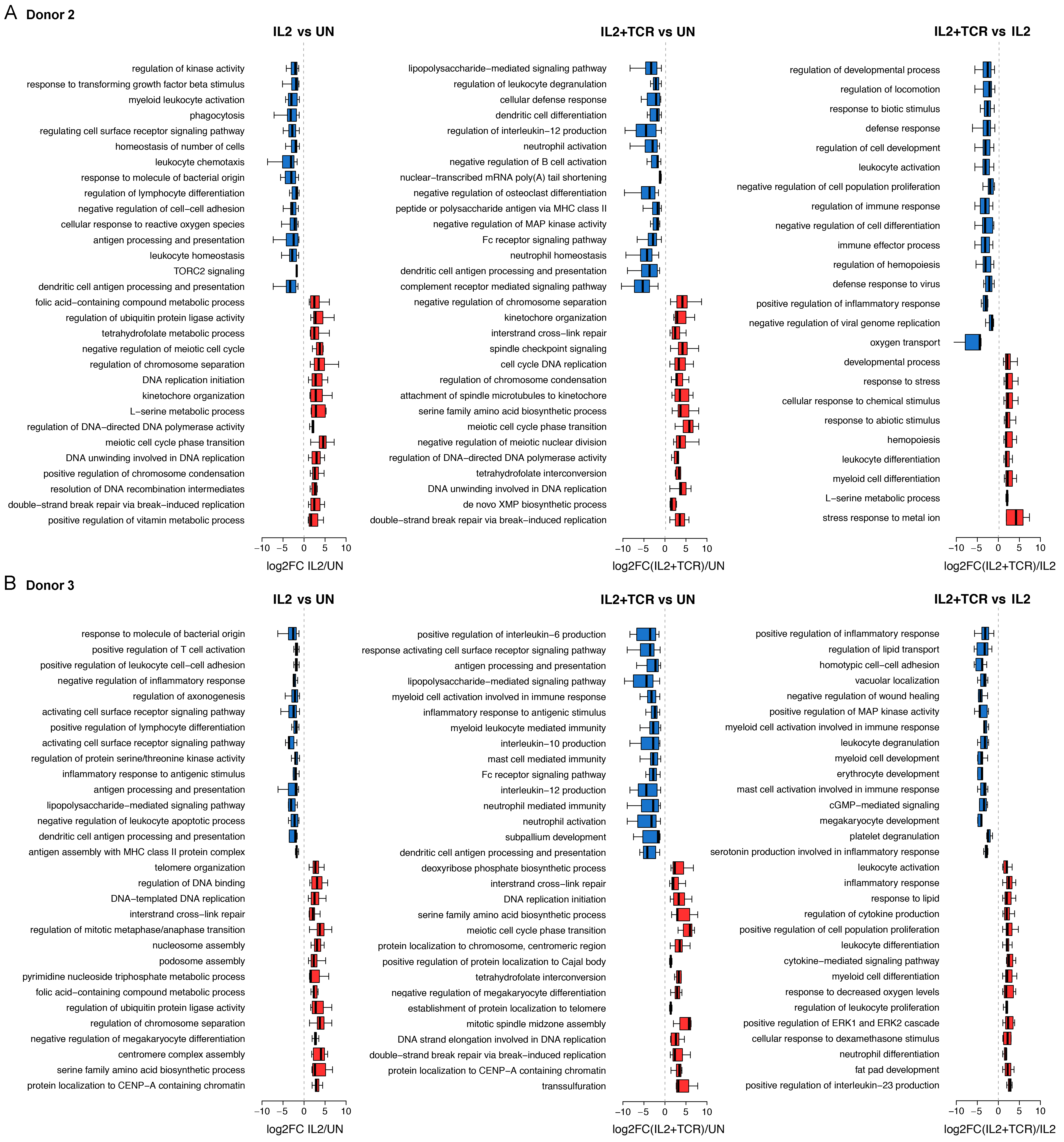
**

**Supplementary Figure S5.** GO-term plots for the 15 most enriched over expected functional terms for genes changing expression in each condition for donors 2 and 3 matched to donor 1 data shown in Figure 2, panel D. **(A)** Donor 2. **(B)** Donor 3.

**
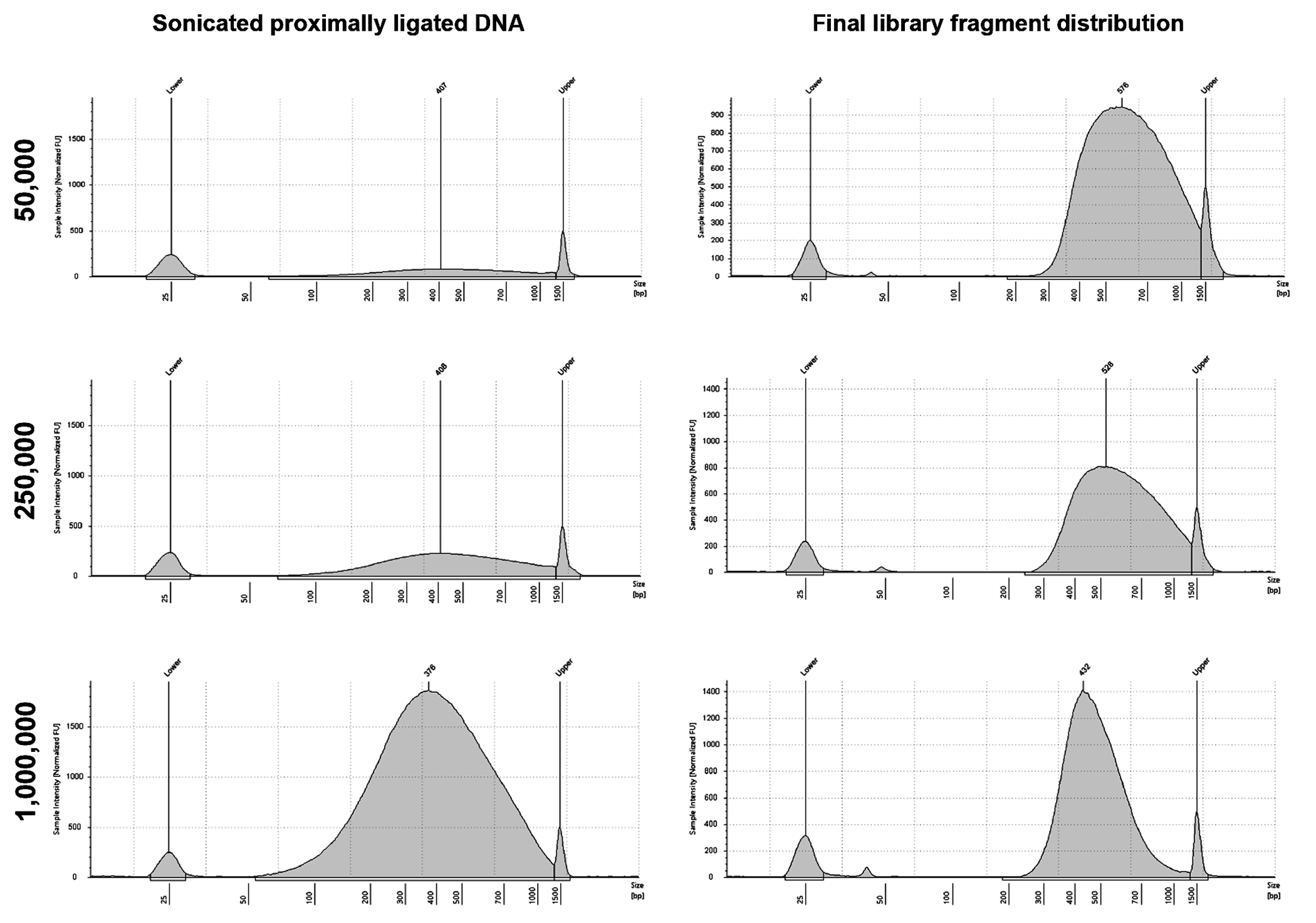
**

**Supplementary Figure S6.** Representative fragment distribution electropherograms (Agilent Tapestation). Fragment distribution of sonicated proximally ligated DNA and the final post-PCR amplification libraries. Plots are taken from libraries generated from donor 1 unstimulated cells with formaldehyde fixed cell number inputs marked on the left. Plots are representative of the rest of the *in situ* Hi-C libraries generated (not shown).

**
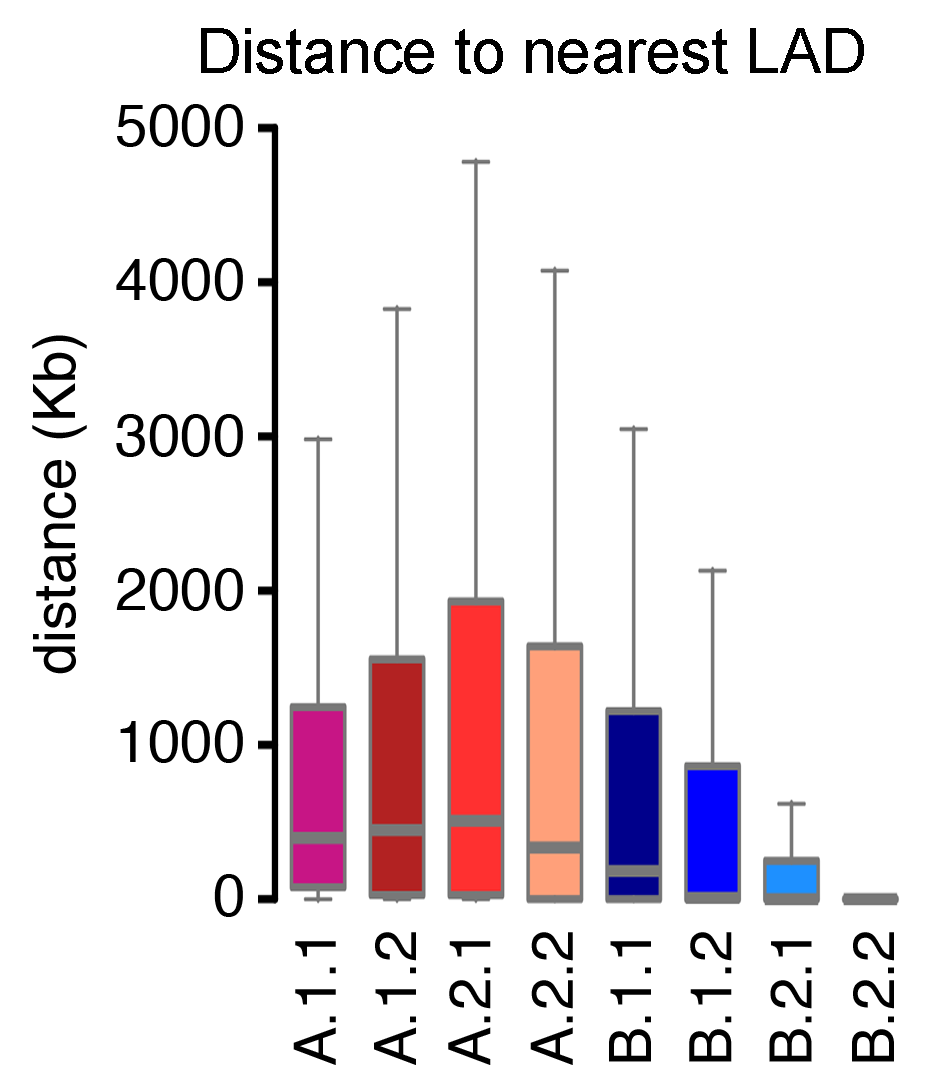
**

**Supplementary Figure S7.** Plot of distance of each CALDER subcompartment to the nearest LAD.

## Supplementary Table Legends

**Supplementary Table S1.** RNA extraction and library details for RNAseq.

**Supplementary Table S2**. RNAseq results. Table showing expression values (FPKM and zFPKM) for each treatment and donor, and the differential gene expression data for each pairwise combination of treatments (log2FC and adjusted p-values: FDR, false discovery rate). The prefix for the differential expression columns indicate the donor and treatment comparison. The first element (d1, d2 or d3) indicate the donor. The second and third elements indicate the comparison in question, with un = unstimulated, il2 = IL2 treatment, and ab = IL2+TCR treatment.

**Supplementary Table S3.** Functional analysis of differentially expressed genes. The differentially expressed gene lists were analysed with g:profiler using the default parameters, full results. A summary is shown in Figure 2D for donor 1, and Supplementary Figure S5 for donors 2 and 3.

**Supplementary Table S4**. HiC library sample details.

**Supplementary Table S5**. A-B and B-A compartment switches for donor1. Genes included in these regions are shown, as well as their log2FC values for the 3 pairwise treatment comparisons in donor 1. The prefix for the differential expression columns indicate the donor and treatment comparison. The first element (d1) indicate the donor. The second and third elements indicate the comparison in question, with un = unstimulated, il2 = IL2 treatment, and ab = IL2+TCR treatment.

**Supplementary Table S6.** Differential TAD analysis for donor 1. HOMER was used to calculate significant changes in TAD inclusion ratios (IR).

**Supplementary Table S7**. Differential loop analysis for donor 1. HOMER was used to calculate significant changes in loop interaction scores.

**Supplementary Table S8.** Enhancer-promoter interactions. Differential E-P interactions were calculated with HOMER after performing a virtual 4C analysis with HiContacts. The column name prefix ‘e’ indicates enhancer, and ‘p’ promoter. Distance.EP indicates the distance (in bp) between enhancer and promoter. Gene names are highlighted in red or blue to indicate an increase or a decrease of interactions. D.nearest.prom indicates the distance of the detected enhancer-interacting region to the nearest promoter (in bp).
